# α-Synuclein promotes IAPP fibril formation in vitro and β-cell amyloid formation in vivo in mice

**DOI:** 10.1038/s41598-020-77409-z

**Published:** 2020-11-24

**Authors:** Marija Mucibabic, Pär Steneberg, Emmelie Lidh, Jurate Straseviciene, Agnieszka Ziolkowska, Ulf Dahl, Emma Lindahl, Helena Edlund

**Affiliations:** 1 Umeå Centre for Molecular Medicine, Umeå University, 901 87 Umeå, Sweden; 2 Department of Chemistry, Umeå University, 901 87 Umeå, Sweden

**Keywords:** Protein aggregation, Type 2 diabetes

## Abstract

Type 2 diabetes (T2D), alike Parkinson’s disease (PD), belongs to the group of protein misfolding diseases (PMDs), which share aggregation of misfolded proteins as a hallmark. Although the major aggregating peptide in β-cells of T2D patients is Islet Amyloid Polypeptide (IAPP), alpha-synuclein (αSyn), the aggregating peptide in substantia nigra neurons of PD patients, is expressed also in β-cells. Here we show that αSyn, encoded by *Snca*, is a component of amyloid extracted from pancreas of transgenic mice overexpressing human *IAPP* (denoted *hIAPPtg* mice) and from islets of T2D individuals. Notably, αSyn dose-dependently promoted IAPP fibril formation in vitro and tail-vein injection of αSyn in *hIAPPtg* mice enhanced β-cell amyloid formation in vivo whereas β-cell amyloid formation was reduced in *hIAPPtg* mice on a *Snca *^*−/−*^ background. Taken together, our findings provide evidence that αSyn and IAPP co-aggregate both in vitro and in vivo, suggesting a role for αSyn in β-cell amyloid formation.

## Introduction

Protein aggregation into amyloid fibrils is a key event in > 30 protein misfolding diseases (PMDs) including type 2 diabetes (T2D)^1,2^; > 90% of all type 2 diabetics have amyloid deposits in at least one islet and in some type 2 diabetics up to 80% of the islets show evidence of amyloid deposits^3–5^. In β-cells of T2D individuals the formation of toxic aggregates of islet amyloid poypeptide (IAPP), also known as amylin, is associated with β-cell stress and β-cell dysfunction and accumulation of IAPP amyloid fibrils provokes β-cell death and development of diabetes^6–8^. Previous work by us and others have shown that alpha-Synuclein (αSyn), the major aggregating protein that forms insoluble fibrils found in Lewy bodies in diseases such as Parkinson's disease (PD) and dementia with Lewy bodies, is expressed also in pancreatic β-cells^9–11^ and that αSyn levels are increased in β-cells of mice lacking the T2D associated gene *Ide*^10^ and in islets from T2D patients^10,11^.

Accumulating data from large prospective cohort studies suggest that T2D and other PMDs such as Alzheimer’s disease (AD) and PD are associated; T2D patients have 50–100% increased risk of developing AD^12,13^ and a ~ 35–40% increased risk of developing PD^14–17^, with some studies reporting ~ 100% increased risk^16,17^, as compared to non-diabetic subjects. One feature shared by T2D, AD, and PD, is the strong association with insulin resistance^14,18,19^. Although the exact underlying cause(s) remains largely unknown, the pathological similarities, i.e. protein aggregation and association with insulin resistance, between T2D, AD, and PD suggest that these conditions share general, disease provoking molecular mechanisms. Insulin resistance provoked hyperglycemia not only leads to endoplasmic reticulum (ER) stress in β-cells, which increases the intracellular amount of unfolded and misfolded proteins, but also to increased levels of intracellular calcium and mitochondrial stress, i.e. conditions that are known to promote αSyn aggregation^20,21^. Notably, amyloidogenic proteins not only have the potential to self-seed into homologous fibrils but can also cross-seed to form heterologous fibrils in vitro^22^. Although Aβ is the main component of Alzheimer’s plaques, both IAPP and an internal region of αSyn, denoted NAC, have been detected in Aβ deposits^23,24^ and Aβ fibrils has been suggested to seed IAPP amyloid^23^, leaving open the possibility of cross-seeding between amyloidogenic peptides also in vivo, which in turn may enhance the onset and/or progression of PMDs.

Here we show that IAPP and αSyn co-localized in β-cell amyloid extracted from *hIAPPtg* mouse pancreases and human β-cells and that αSyn enhanced IAPP fibril formation in vitro in a dose-dependent manner. We also show that β-cells internalized exogenously administered αSyn and that tail-vein injection of αSyn into *hIAPPtg* mice enhanced β-cell amyloid formation whereas amyloid formation was reduced in *hIAPPtg* mice on an *Snca*^*−/−*^ background. Together, our findings provide evidence for a role for αSyn in IAPP aggregation and β-cell amyloid formation.

## Results

### αSyn and IAPP co-localize in mouse and human β-cells and islet amyloid

Rodent IAPP is not amyloidogenic^25^, thus to explore a potential functional association between IAPP and αSyn during β-cell amyloid formation we made use of a transgenic mouse T2D model that express *human IAPP* (*hIAPP*) in β-cells under the control of the *rat insulin 2 promoter*, denoted *hIAPPtg* mice. Electron microscopy (TEM) analyses of double αSyn and IAPP immunogold labelled islets isolated from *hIAPPtg* mice and T2D individuals showed that, as previously described using the proximity-ligation-assay on human pancreatic sections^11^, αSyn and IAPP co-exists in close proximity in β-cells (Supplementary Fig. 1a-h). αSyn immunoreactivity was not observed when staining islets isolated from *hIAPPtg* mice on a *Snca*^*−/−*^ backgound, demonstrating the specificity of the αSyn antibodies (Supplementary Fig. 2a,b).

**Figure 1:**
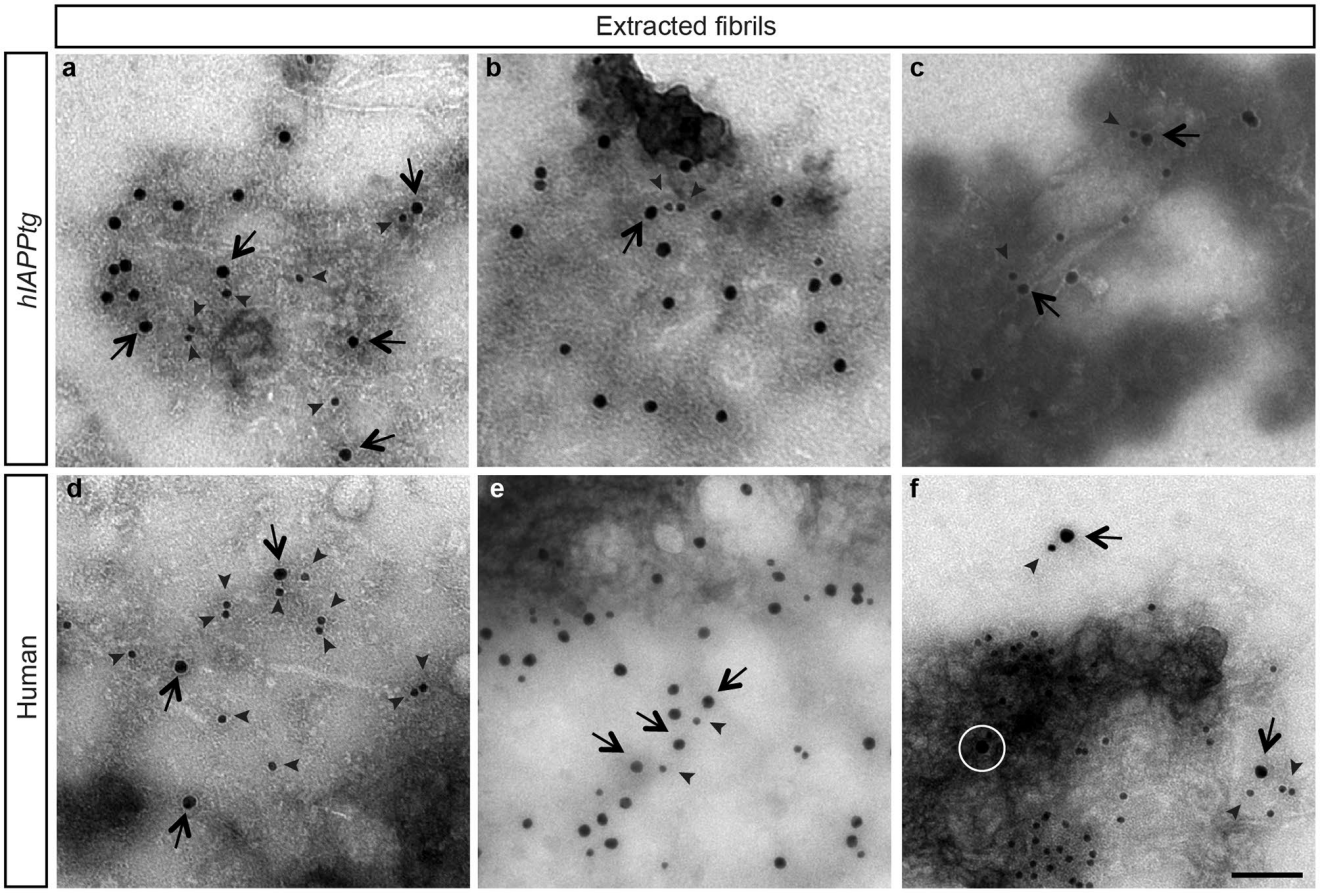
αSyn and IAPP co-localize in mouse and human islet amyloid. **(a-f)** TEM images of fibrils extracted from 3 independently pooled *hIAPPtg* mouse pancreases, 2 pooled pancreases/extract, **(a-c),** and human islets **(d-f)** (donors #4, 5, and 6 from left to right) showing immuno-gold labelled αSyn (sc-7011R, 15 nm gold particles) and IAPP (NBP1-06579, gold 10 nm gold particles). Black arrows and white circle indicate αSyn and arrow heads indicate IAPP labelled gold particles. Scale bar is 100 nm in (**a–f)**.

**Figure 2:**
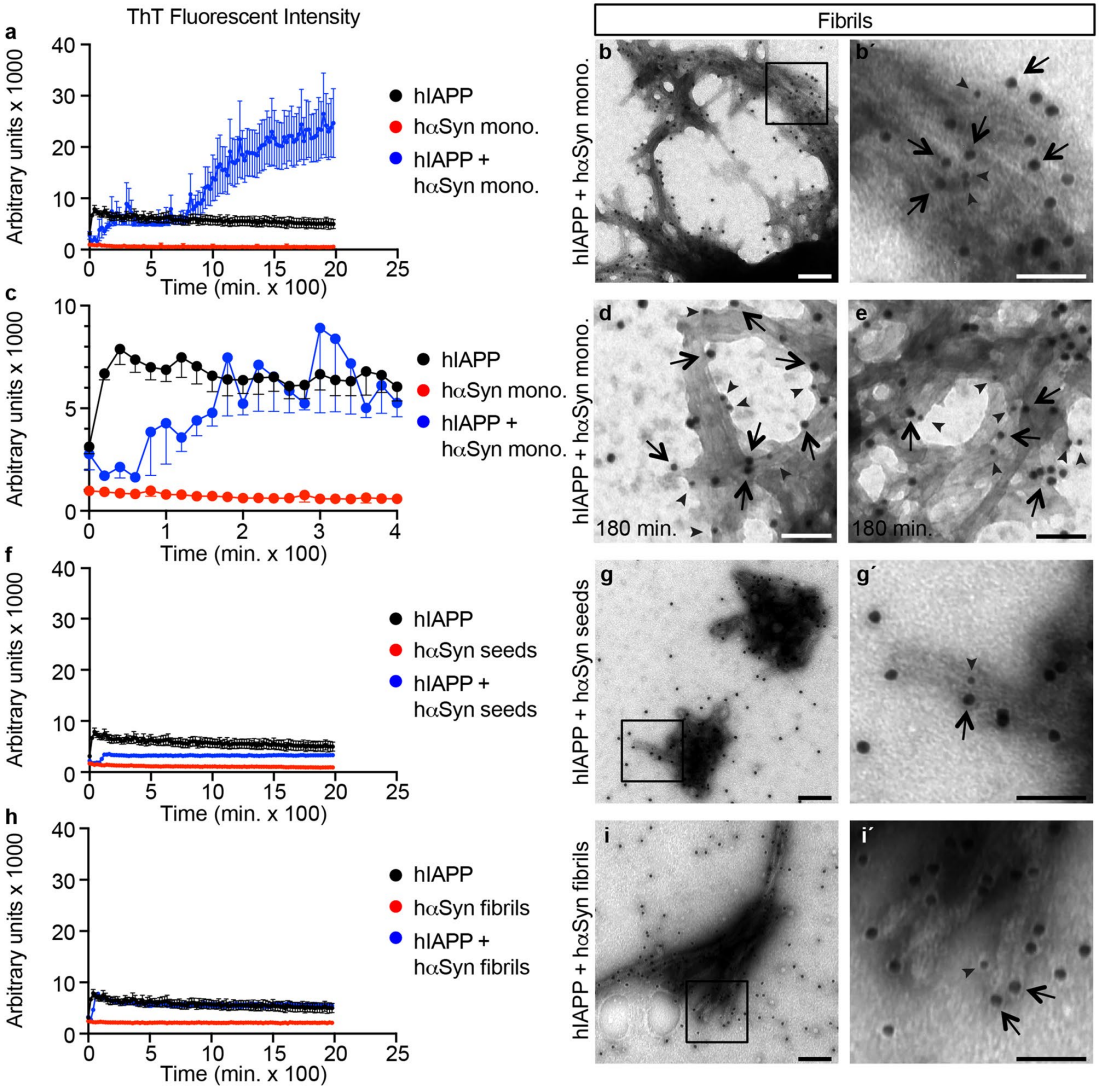
α-Syn monomers cross-seed IAPP fibril formation. (**a, c**) Fibril formation ThT curves for the entire 2000 min incubation period (**a**) and a close up of the first 400 min (**c**) for 2 µM hIAPP monomers alone (black), 7 µM hαSyn monomers alone (red), and 2 µM hIAPP monomers with 7 µM hαSyn monomers (blue). (**b,b’,d,e**) TEM images of immuno-gold labelling for αSyn (sc-7011R, 15 nm gold particles) and IAPP (NBP1-06579, 10 nm gold particles) of the resulting fibrils at 2000 min (**b,b’**) and at 180 min (**d,e**) from incubating 2 µM hIAPP monomers with 7 µM hαSyn monomers. (**b’**) show marked area (open square) in (**b)** in higher magnification. (**f,h**) Fibril formation ThT curves for 2 µM hIAPP monomers alone (black in **f,h**), 7 µM hαSyn seeds alone (red in **f**), 7 µM hαSyn fibrils alone (red in **h**), and 2 µM hIAPP monomers with 7 µM hαSyn seeds (blue in **f**) and 7 µM hαSyn fibrils (blue in **h**), respectively. (**g,g’,i,i’**) TEM images of immuno-gold labelling for αSyn (sc-7011R, 15 nm gold particles) and IAPP (NBP1-06579, 10 nm gold particles) of the resulting fibrils (end-stage) from incubating 2 µM hIAPP monomers with 7 µM hαSyn seeds (**g**) and fibrils (**i**), respectively. (**g’,i’**) show marked areas (open square) in (**g**), and (**i),** respectively, in higher magnification. Black arrows indicate αSyn and arrow heads indicate IAPP labelled gold particles. Data in **(a,c,f,h)** are presented as mean value + /− SEM. Scale bar is 200 nm in (**b,g,i)** and 100 nm in (**b’,d,e,g’,I’)**.

To elucidate whether αSyn not only is expressed in β-cells but also might constitute part of β-cell amyloid, we extracted amyloid from pancreases of *hIAPPtg* mice and isolated human islets from T2D individuals. Double anti-αSyn and anti-IAPP TEM immunogold analyses of extracted amyloid showed that not only IAPP but also αSyn were present in amyloid fibrils extracted from *hIAPPtg* pancreas (Fig. 1a–c) and human islets (Fig. 1d–f). Together, these findings show that αSyn not only is expressed in β-cells but that αSyn also is a component of the amyloid formed in β-cells of a T2D mouse model and T2D human subjects.

### αSyn promote IAPP fibril formation in vitro

Previous work have shown that IAPP and αSyn can cross-react in vitro^26^. To investigate whether IAPP and αSyn in vitro cross-seeding results in the formation of hybrid amyloid fibrils, as suggested by the presence of αSyn in extracted β-cell amyloid (Fig. 1), 7 µM human αSyn (hαSyn ) monomers were co-incubated with 2 µM human IAPP (hIAPP) monomers. Amyloid fibril formation was monitored through Thioflavin T (ThT) emission, which measures the specific binding of ThT to formed β-sheets of amyloid fibrils and thus can be used as a proxy for the amount of amyloid fibrils formed^27^. The low (2 µM) hIAPP monomer concentration was chosen since hIAPP homoaggregation is very rapid with reported fibrillar growth observed already within 5–10 minutes (min) of incubation^26^. Consistently, hIAPP homo-seeding showed a very short lag phase of ~ 5 min (Supplementary Fig. 3), followed by a brief elongation phase with a low final maximum ThT emission plateau already after ~ 30 min (Supplementary Fig. 3). At the 7 µM concentration hαSyn monomers alone did not form fibrils as judged from the lack of ThT emission (Fig. 2a,c).

**Figure 3:**
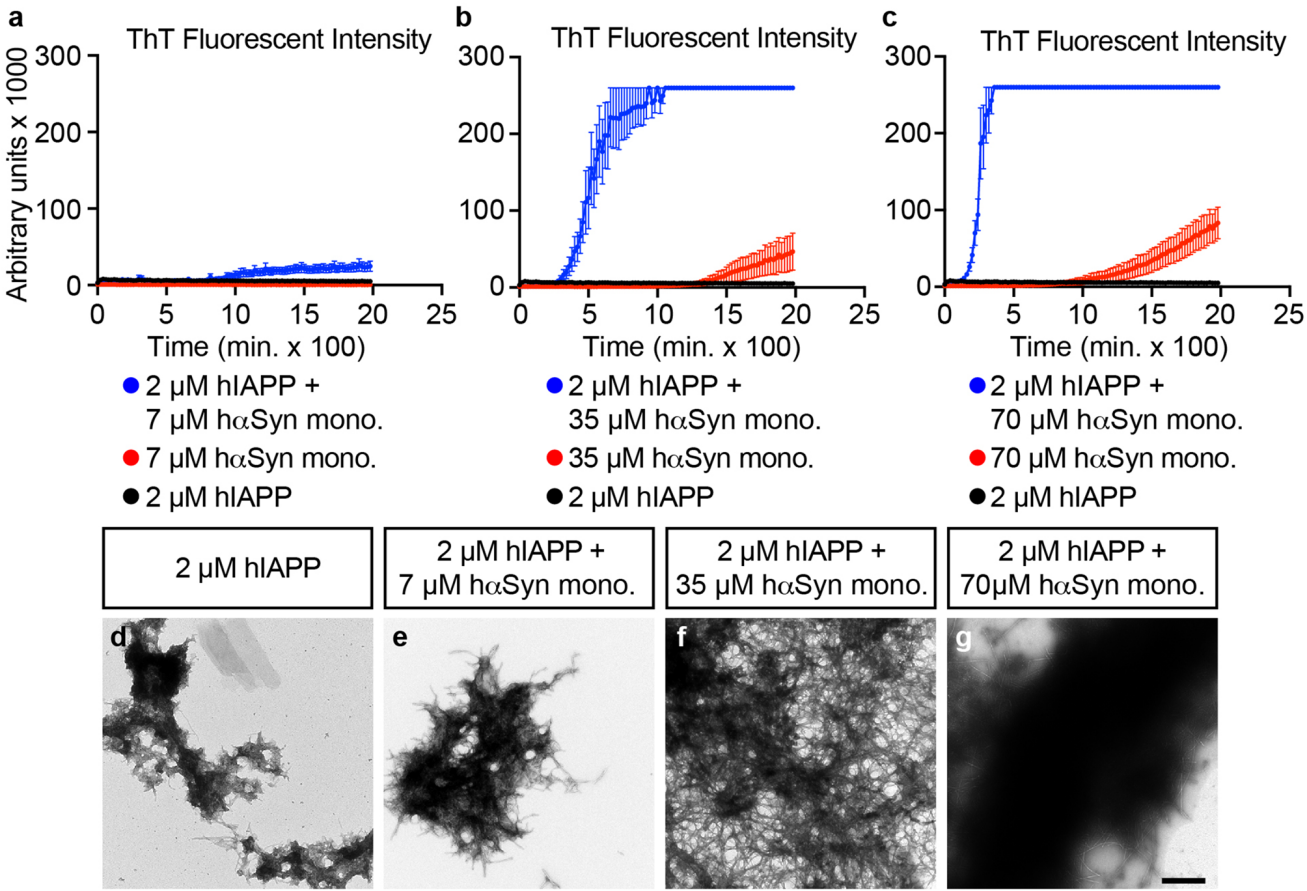
αSyn monomers dose-dependently cross-seed IAPP fibril formation. (**a–c**) Fibril formation ThT curves for 2 µM hIAPP monomers alone (black) and with 7, 35, and 70 µM hαSyn monomers (blue), shown are also curves for 7, 35, and 70 µM hαSyn monomers alone (red). Please note that (**a)** is adapted from Fig. 2a but presented with a different scale on the x-axis to allow direct comparison with ThT emissions curves in (**b,c).** Also**,** please note that the ThT emission exceeds the setting of the plate reader when co-incubating 2 µM hIAPP monomers with 35 and 70 µM hαSyn monomers (blue curves in **b,c**), hence the maximal ThT emissions for these reactions are likely larger than that displayed in (**b,c)**. Data in **(a–c)** are presented as mean value + /− SEM. (**d–g**) TEM negative stain images of the resulting fibrils formed from 2 µM hIAPP alone (**d**), 2 µM hIAPP with 7 µM (**e**), 35 µM (**f**), and 70 µM (**g**) hαSyn, respectively. Scale bar is 400 nm in (**d–g)**.

Co-incubation of 2 µM hIAPP monomers with 7 µM hαSyn monomers resulted in fibrillar growth as evident by the increase in ThT emission intensities (Fig. 2a) and the increase in αSyn and IAPP positive pelletable aggregates isolated from the first lagphase to the end platueau (Supplementary Fig. 4). The maximum ThT emission observed at the end of the reaction for co-incubated hIAPP and hαSyn was ~ fivefold higher than that observed for hIAPP alone (Fig. 2a), suggesting that hIAPP and hαSyn cross-seed fibril formation. Consistently, hαSyn and IAPP TEM double immunogold labelling of the end-stage formed fibrils revealed prominent, dense fibrillar structures that was composed of both αSyn and hIAPP, suggesting that under these conditions hαSyn and hIAPP form heterofibrils (Fig. 2b,b’). In contrast, co-incubation of non-amyloidogenic rat IAPP monomers with hαSyn monomers did not result in fibrillar growth (Supplementary Fig. 5). Taken together, these data provide evidence that hαSyn and hIAPP monomers cross-seed amyloid fibril formation and that hαSyn and hIAPP monomers co-aggregate to form hybrid amyloid fibrils.

**Figure 4:**
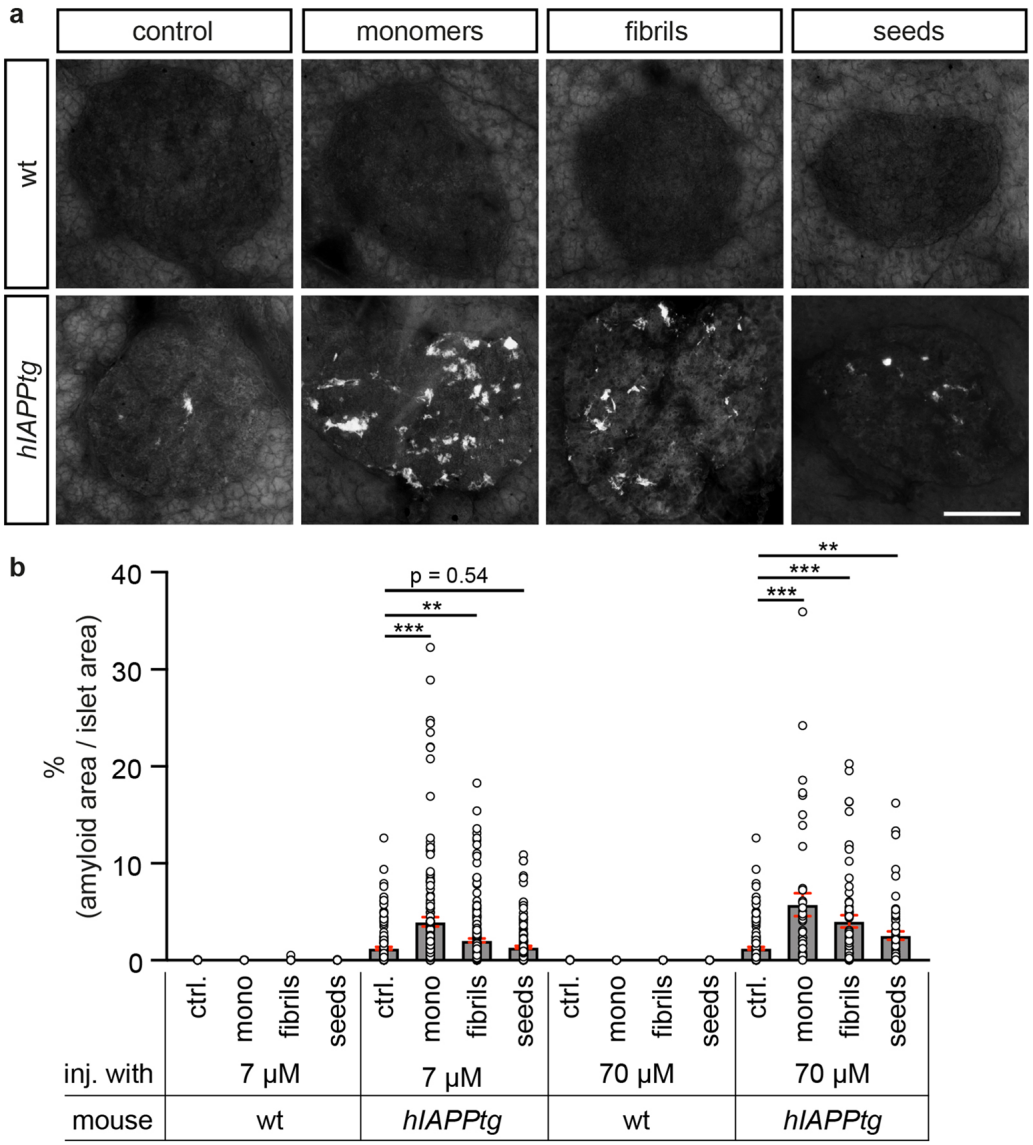
Tail vein injection of αSyn enhances amyloid formation in *hIAPPtg* mice. Islet amyloid quantification in pancreas from tail vein injected *hIAPPtg* mice. (**a**) Representative images of Thio-S^+^ amyloid deposits in islets of wild type (wt; upper panel) and *hIAPPtg* (lower panel) mice tail vein injected with PBS (= control), and 7 µM of hαSyn monomers, fibrils or seeds, respectively. (**b**) Quantification of percentage Thio-S^+^ amyloid area/islets area of wild type and *hIAPPtg* mice tail vein injected with PBS (= ctrl.), 7 and 70 µM hαSyn monomers, fibrils or seeds, respectively. Please see “Amyloid analyses” in the Materials and methods section for information on numbers of islets and mice analysed for each category. Scale bar is 100 µm. Data are presented as mean + /− SEM, **P < 0.01, ***P < 0.001 (Student’s t-test).

**Figure 5:**
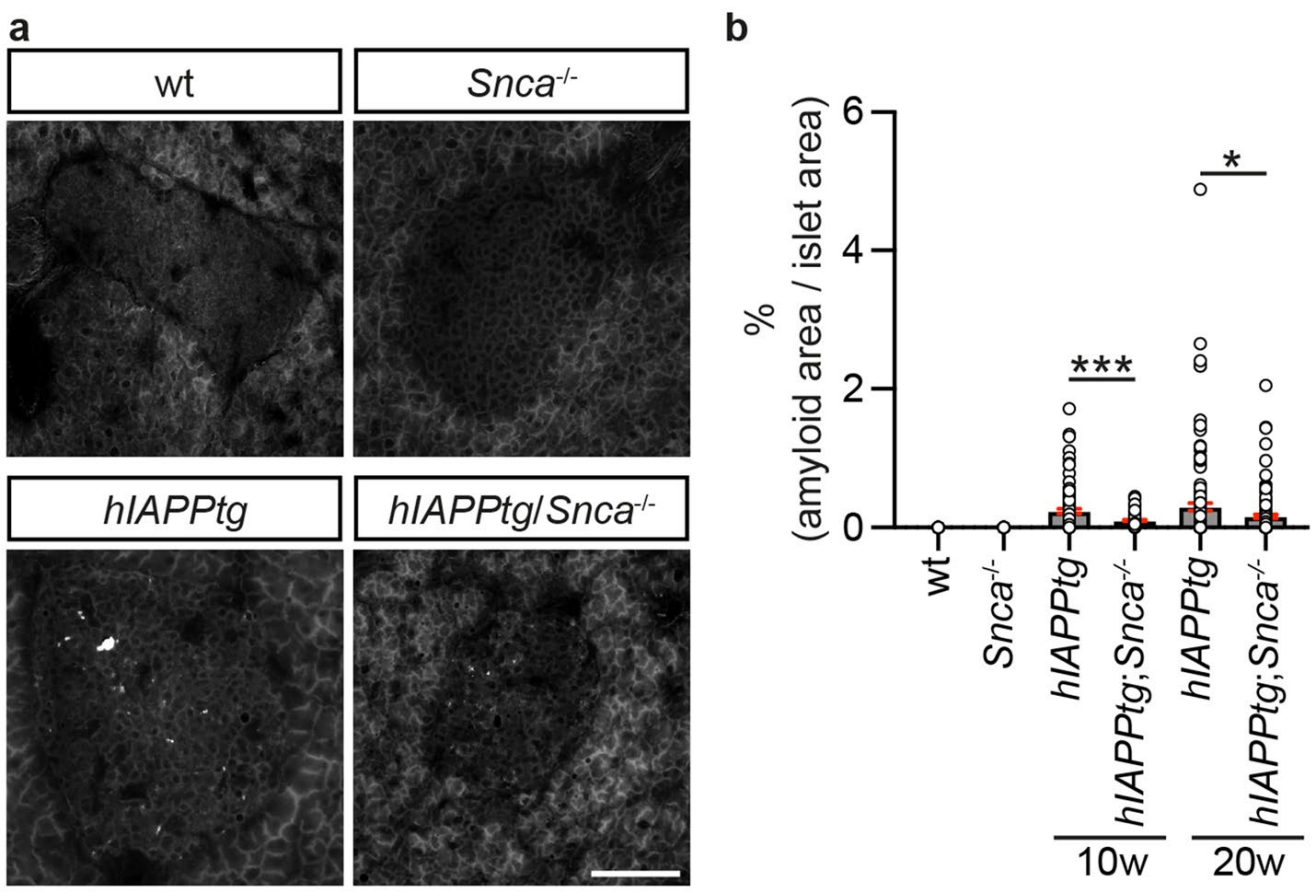
Reduced amyloid formation in *hIAPPtg* mice devoid of endogenous αSyn. **(a)** Representative images of Thio-S^+^ amyloid deposits in islets from 10 weeks old wild type (wt), *Snca*^*−/−*^, *hIAPPtg*, and *hIAPPtg;Snca*^*−/−*^ mice. (**b**) Quantification of percentage Thio-S^+^ amyloid area/islets area from 10 weeks old wt and *Snca*^*−/−*^ mice and 10 and 20 weeks old *hIAPPtg* and *hIAPPtg;Snca*^*−/−*^ mice. Please see “Amyloid analyses” in the Materials and methods section for information on numbers of islets and mice analysed for each category. Scale bar is 100 µm**.** Data are presented as mean + /- SEM, *P < 0.05, ***P < 0.001.

Cross-seeding 2 µM hIAPP monomers with 7 µM hαSyn monomers, resulted in atypical kinetics with a long, ~ 80 min, lag/nucleation phase (Fig. 2a,c), followed by a ~ 100 min elongation phase, a second longer ~ 680 min lag phase, and a final, ~ 20 h long elongation phase (Fig. 2a). The atypical kinetics observed when co-incubating 2 μM hIAPP with 7 μM hαSyn monomers is intriguing and suggest that, alike that described for αSyn homoseeding at low concentrations^28^, during the first elongation phase hIAPP and hαSyn form stable prefibrillar amyloid aggregates capable of binding ThT and that these prefibrillar amyloid aggregates seeds/nucleates further cross-seeding into mature fibrils during the second elongation phase. αSyn and IAPP double immuno gold labelling of samples collected already after 3 h of cross-seeding 2 μM hIAPP with 7 μM hαSyn monomers, i.e. at the end of the first elongation phase (Fig. 2c), demonstrated the presence of both hαSyn and hIAPP in the by then formed aggregates (Fig. 2d,e), providing evidence for the formation of pre-fibrillar hetero-aggregates during the first elongation stage.

Next 7 µM hαSyn seeds, i.e. sonicated pre-formed hαSyn fibrils (Supplementary Fig. 6), and hαSyn pre-formed fibrils, respectively, were co-incubated with hIAPP monomers. Compared with that of 2 µM hIAPP monomers alone, 7 µM hαSyn seeds negatively influenced amyloid fibril formation (Fig. 2f) and hαSyn pre-formed fibrils resulted in ThT emission curves that were superimposed over that of 2 µM hIAPP monomers alone (Fig. 2h). Double αSyn and IAPP immuno gold TEM analyses revealed that the fibrils formed when combining hαSyn pre-formed seeds or fibrils with hIAPP monomers were relatively short and sparse and that the fibrils consisted predominantly of αSyn with occasional IAPP (Fig. 2g,g’,i,i’). Together, these data suggest that at equimolar concentrations to that of monomers, i.e. 7 µM, pre-formed seeds and fibrils do not promote fibrillar growth of hIAPP monomers nor hIAPP hetero-amyloid fibril formation.

**Figure 6:**
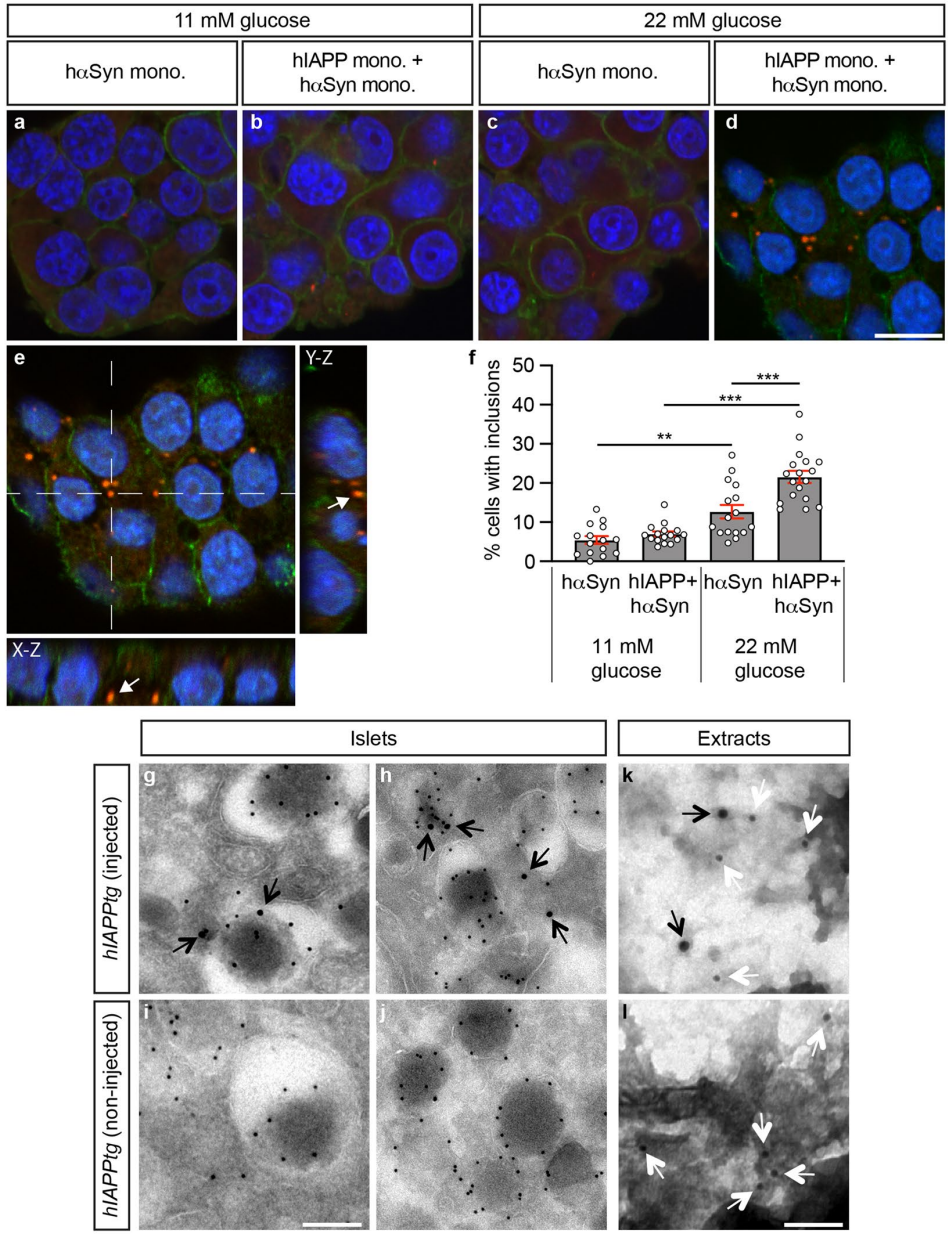
Exogenously administered αSyn is internalized into β-cells. (**a–e**) Confocal images (**a–d**) and confocal x–y–z stacks image (**e**) of the internalization of αSyn in INS-1E cells incubated with 1 μM hαSyn momomers for 24 h (**a,c**) or 5 μM hIAPP monomers for 4 h followed by 1 μM hαSyn monomers for 20 h (**b,d,e**) at 11 (**a,b**) and 22 mM (**c–e**) glucose, respectively, and immunostained with human specific αSyn antibodies (sc-12767; red) and Syntaxin antibodies (sc-13994; green). The crossed lines in (**e**) indicate the position of a cytoplasmic α-Syn inclusion in the x–z and y–z planes. (**f**) Quantification of αSyn^+^ inclusions in INS-1E cells incubated with 1 μM hαSyn momomers for 24 h or 5 μM hIAPP monomers for 4 h followed by 1 μM hαSyn monomers for 20 h at 11 and 22 mM glucose, respectively. Data in **f** are presented as mean value + /− SEM. (**g–l**) TEM images of islets isolated from independent mice (**g–j**) and extracted amyloid (from 2 pooled, independent pancreases, respectively) (**k,l**) of *hIAPPtg* mice tail vein injected with 70 µM hαSyn monomers (**g,h,k**) and non-injected controls (**i,j,l**), immuno-gold labelled with human specific αSyn [sc-12767 (**g–l**), 15 nm gold particles in **g,h,k**], IAPP (NBP1-06579, 10 nm gold particles in **g–j**), and pan-αSyn (sc-7011R, 10 nm gold particles in **k,l**) antibodies. Black arrows indicate human specific αSyn and white arrows indicate pan-αSyn labelled gold particles. Scale bar is 10 µm in (**a–d)**, 200 nm in (**g–j)** and 100 nm in (**k,l)**. Data are presented as mean + /− SEM, **P < 0.01, ***P < 0.001 (Student’s t-test).

### αSyn monomers dose-dependently stimulate IAPP fibril formation in vitro

To further elucidate the cross-seeding potential of hαSyn and hIAPP monomers, 2 μM hIAPP monomers were incubated with increasing concentrations of hαSyn monomers. Alike the combination of 2 μM hIAPP and 7 μM hαSyn monomers, (Figs. 2a,3a; please note the difference in scale on the y-axis between Figs. 2a and 3a), higher concentrations, 35 and 70 μM, of hαSyn monomers combined with 2 μM hIAPP monomers also delayed the nucleation/lag phase compared with that of hIAPP alone (Supplementary Fig. 7). There was however a does-dependent decrease in lag phases when cross-seeding hIAPP with higher concentrations of hαSyn monomers, i.e. with the lag phases at 70 μM < 35 μM < 7 μM (Fig. 3a–c). Moreover, the end stage ThT emission when cross-seeding IAPP monomers with 35 and 70 μM of hαSyn monomers was not only distinctly higher than that observed when co-incubating 2 μM hIAPP and 7 μM hαSyn monomers (Fig. 3a–c) but resulted in maximal measurable ThT emission levels that exceeded the setting of the plate reader (Fig. 3b,c). TEM negative stain analyses of end-stage reactions showed formation of fibrils with an inceasing density that paralleled the increased hαSyn monomer concentration used in the reactions (Fig. 3d–g). Similarly, when using a higher hIAPP monomer concentration of 4 μM, co-incubation with 7, 35, and 70 μM hαSyn monomers potently and dose dependently cross-seeded fibril formation as evident from the increase in both ThT emission (Supplementary Fig. 8a) and fibrillar density (Supplementary Fig. 8b–e). Reciprocally, hIAPP monomers in concentrations of 2, 4 and 8 μM, enhanced ThT emission of 70 μM hαSyn monomers and also resulted in the formation of fibrillar tangles with increasing density (Supplementary Fig. 9a–e). Taken together, these findings, provide evidence that hαSyn and hIAPP monomers dose-dependently cross-seed fibrillar growth.

Although 7 μM hαSyn seeds and fibrils did not stimulate fibril growth when combined with 2 μM hIAPP monomers (Fig. 2f,h), higher concentrations, i.e. 35 and 70 μM, of hαSyn seeds and fibrils enhanced ThT emission levels as compared with that of hIAPP monomers or hαSyn seeds and fibrils alone (Supplementary Fig. 10a–f). The resulting ThT emissions when cross-seeding IAPP monomers with higher concentrations of hαSyn seeds and fibrils were however considerably lower than that observed when combining equimolar concentrations, i.e. 35 and 70 μM, of hαSyn monomers with hIAPP monomers (note the difference in scales in Fig. 3a–c compared with those in Supplementary Fig. 10a–f). Cross-seeding 2 μM hIAPP monomers with lower, 0.2 μM, concentrations of hαSyn monomers, seeds, and fibrils resulted in none to very limited increase in ThT emission as compared to that of hIAPP monomers alone (Supplementary Fig. 11a–c). Together, these data provide evidence that hαSyn and hIAPP monomers dose-dependently and bi-directionally cross-seed and co-aggregate into hybrid amyloid fibrils at concentrations of αSyn monomer of 7 μM or higher, whereas seeds and fibrils are less potent at cross-seeding IAPP monomer fibril formation and growth at equimolar concentrations.

### αSyn promotes islet amyloid formation in *hIAPPtg* mice in vivo

To explore whether αSyn may enhance IAPP amyloid formation also in vivo*,* hαSyn monomers, fibrils, and seeds were intravenously (i.v.) injected via the tail vein into 10 weeks old *hIAPPtg* and wild type (wt) litter mate controls. 10 weeks following the injection, mice were sacrificed and the amount of pancreatic islet amyloid quantified. Compared with *hIAPPtg* mice injected with PBS, % average amyloid area/islet area was increased, by 225%, in *hIAPPtg* mice injected with 7 μM hαSyn monomers and by 370% in *hIAPPtg* mice injected with 70 μM hαSyn monomers (Fig. 4b), indicating a dose dependent increase in islet amyloid in response to injection of hαSyn monomers. Injection of fibrils or seeds also increased islet amyloid formation compared with that of PBS injected *hIAPPtg* mice, albeit less potently than hαSyn monomers; injection of 7 μM hαSyn fibrils increased % average amyloid/islet area by 70% whereas injection of 7 μM hαSyn seeds showed a non-significant (p-value = 0.54) increase in % average amyloid/islet area (Fig. 4a,b). Alike that observed for hαSyn monomers, injection of a higher concentration, 70 μM, of hαSyn fibrils and seeds resulted in further % average amyloid/islet area, now by 240% and 110%, respectively, compared with that of *hIAPPtg* mice injected with PBS (Fig. 4b). Thus, hαSyn species dose-dependently, and monomers more potently than seeds and pre-formed fibrils, promoted IAPP amyloid fibril formation also in vivo. Notably, no islet amyloid was detected in wt mice injected with the various hαSyn species, demonstrating that the exogenously administered hαSyn is not capable of generating islets amyloid deposits by itself but is dependent on, and accelerates, hIAPP misfolding and fibril formation in β-cells of *hIAPPtg* mice.

Next, *hIAPPtg* mice were crossed onto a *Snca*^*−/−*^ background, i.e. mice lacking endogenous αSyn, and the amount of islet amyloid formed in *hIAPPtg* and *hIAPPtg; Snca*^*−/−*^ pancreases was quantified. As expected, no amyloid was detected in islets of either wt or *Snca*^*−/−*^ mice (Fig. 5a,b). Notably, a significant reduction of % average amyloid/islet area was observed in pancreases of *hIAPPtg/Snca*^*−/−*^ mice as compared with that of *hIAPPtg* mice with a 37% and 59% reduction in % average amyloid/islet area at 10 and 20 weeks of age, respectively (Fig. 5a,b), providing evidence that endogenous αSyn contribute to hIAPP amyloid formation in vivo in β-cells of *hIAPPtg* mice. Taken together, these results provide strong evidence that αSyn potently cross-seeds hIAPP fibril formation and thus β-cell amyloid formation in vivo.

### Exogenously administered αSyn momomers are internalized into β-cells and islet amyloid

Although amyloid deposits of T2D islets are extracellular with the potential to seed further amyloid growth, amyloid formation is thought to be initiated intracellularly^8^, raising the notion that tail vein injected αSyn may stimulate amyloid formation in *hIAPPtg* mice intracellularly. To address a potential intracellular mode of action for exogenously administered αSyn we exposed a rat β-cell line, INS-1E, to hαSyn momomers. Treatment of INS-1E cells cultured at 11 mM glucose with either 1 μM hαSyn monomers for 24 h (h) or 5 μM hIAPP monomers for 4 h followed by a 20 h exposure to 1 μM hαSyn monomers resulted in limited internalization of hαSyn but there was a non-significant trend to increased number of hαSyn^+^ inclusions in cells sequentially exposed to hIAPP and hαSyn monomers (Fig. 6a,b,f). hαSyn^+^ inclusions were significantly more prominent when INS-1E cells were cultured at 22 mM glucose than at the 11 mM glucose concentration (Fig. 6a–f). The number of hαSyn^+^ inclusions were also significantly more numerous when INS-1E cells cultured at 22 mM glucose were sequentially exposed to hIAPP and hαSyn monomers as compared with that of cells exposed hαSyn monomers alone (Fig. 6c,d,f). Together these findings provide evidence of an hIAPP and glucose concentration mediated uptake and aggregation of hαSyn into β-cells.

In further support of a potential intracellular cross-seeding scenario mediated by tail vein injected hαSyn, double TEM immunogold labelling analyses of islets isolated from *hIAPPtg* mice injected with 70 μM hαSyn monomers using IAPP and human specific αSyn antibodies revealed the presence of injected hαSyn within β-cells of these mice whereas only IAPP immunoreactivity was observed in islets isolated from non-injected *hIAPPtg* mice (Fig. 6g–j). Double immunogold labelling using human specific αSyn and pan-αSyn antibodies also detected hαSyn in amyloid extracted from αSyn tail-vein injected *hIAPPtg* mice whereas amyloid extracted from non-injected *hIAPPtg* mice only showed immunoreactivity for pan-αSyn antibodies (Fig. 6k,l). Taken together, these data suggest that, although indirect effects of exogenously injected hαSyn can not be excluded, the enhanced amyloid formation observed in *hIAPPtg* mice tail vein injected with hαSyn may result from cross-seeding that at least in part may occur intracellularly.

## Discussion

PMDs currently encompass a long list of disorders that share the aggregation and accumulation of misfolded proteins or peptides into amyloid in distinct cells and organs with cell and organ failure as a consequence^1,2^. Accumulating data suggest that misfolded proteins/peptides not only can self-seed but also cross-seed fibril formation in vitro^22^ but whether cross-seeding actually occurs in vivo remains an open question. Here we show that, although the lag phase is increased compared to that of hIAPP monomers alone, hαSyn monomers at concentrations of 7 μM and higher enhance the fibril elongation phase and the amount of fibrils formed, i.e. as judged from the increased ThT emission observed and TEM analyses of end stage fibrils formed, when co-incubated with hIAPP monomers in vitro. Moreover, double immunogold labelling revealed that the resulting fibrils were composed of both peptides, providing further support for cross-seeding potentials of αSyn and IAPP. The ThT emission curves and TEM analyses of the end stage fibrils provide evidence that the enhancing effect of αSyn monomers is concentration dependent; when combined with a fixed concentration of IAPP, the higher the concentration of αSyn monomers used the more rapid was initiation of fibril formation and the more fibrils appeared to form. Moreover, αSyn monomers not only dose-dependently enhanced the ThT emission intensity compared with that observed with either monomer alone but also provoked the formation of more dense fibrillar structures. At all concentrations tested, αSyn fibrils and seeds were less potent than αSyn monomers in cross-seeding IAPP aggregation.

The atypical aggregation kinetics observed when combining 2 µM hIAPP monomers with 7 µM hαSyn monomers, and that of 4 µM hIAPP monomers with 7 and 35 µM hαSyn monomers, suggest the involvement of distinct growth mechanisms/phases during the fibril formation process, i.e. an intial short lag phase followed by limited growth, followed by a second longer lag phase that finally was followed by a longer, more distinct elongation/growth phase. The mechanisms behind the lag and growth phases observed at these concentrations of hIAPP and hαSyn monomer cross seeding will require more detailed analyses but may resemble that described for homoseeding of low amounts of αSyn monomers^28^, i.e. that an intermediate state of pre-fibrillar aggregates form during the first elongation phase that then seeds the further formation of mature fibrils during the second elongation phase. The double immuno gold labelling of TEM samples collected at the end of the first elongation phase for 2 µM hIAPP monomers with 7 µM hαSyn monomers, showed that both αSyn and IAPP were present in the by then formed fibrils, suggesting that a sufficient proportion of both peptides exhibited conformations favouring co-aggregation and heterofibril formation at early stages. Notably, cross-seeding of IAPP with Aβ 40/42 has identified single hydrophobic/aromatic amino acids within the IAPP amyloid core region that are key for cross-seeding but not IAPP homo-seeding^29^. Morover, studies of Aβ/αSyn cross-seeding has suggested a potential role for the hydrophobic domains of Aβ and the αSyn-NAC region^30^, leaving open the possibility that the aromatic/hydrophobic residues of IAPP and the αSyn-NAC hydrophobic region may mediate IAPP/αSyn heteroaggregation.

The cross-seeding effect of αSyn monomers in promoting IAPP amyloid formation was also evident from the in vivo analyses where a single tail-vein injection of hαSyn monomers significantly enhanced amyloid formation in islets of *hIAPPtg* mice. Moreover, the stimulatory effect of αSyn monomers on IAPP amyloid formation in vivo was dose-dependent; injection of 70 μM hαSyn monomers resulted in a further 64% increase in the amount of amyloid formed as compared with that observed when injecting 7 µm hαSyn monomers. Notably, no amyloid was detected in islets of wt mice injected with the different hαSyn species, arguing against a mere deposition of aggregating hαSyn monomers or pre-formed fibrils and seeds as an explanation for the increased amount of islets amyloid observed in tail-vein injected *hIAPPtg* mice. Thus, the enhanced β-cell amyloid formation observed in tail-vein injected mice is dependent on both endogenous transgene expression of human IAPP and exogenously administered αSyn, providing further argument for a potent cross-seeding and co-aggregation of IAPP and αSyn in vivo.

The increased amount of amyloid formed in hαSyn injected *hIAPPtg* mice could theoretically result from αSyn aggregating on pre-formed, extracellularly deposited IAPP amyloid. The observation that tail vein injected hαSyn was detected not only in extracted amyloid but also within β-cells of injected *hIAPPtg* mice leave open the possibility that αSyn, at least in part, seeds IAPP amyloid formation intracellularly. The uptake and apparent formation of distinct, hαSyn immunoreactive inclusions in INS-1E cells sequentially exposed to hIAPP and hαSyn monomers, provides further support for αSyn internalization and thus potential intracellular cross-seeding of IAPP amyloid formation by αSyn within β-cells. The observation that IAPP appears to prime and/or facilitate αSyn uptake into INS-1E cells together with the observation that the higher 22 mM glucose concentration appears to enhance αSyn uptake and/or aggregation as compared with that observed at the lower 11 mM glucose concentration is intriguing. These findings imply that hIAPP monomers, alike that described for hIAPP oligomers^31^, has the capacity to permeate β-cell membranes or, alternatively, that the exogenously added hIAPP monomers either self oligomerize or bind to the β-cell membrane and then subsequently oligomerize with the sequentially added hαSyn monomers, resulting in β-cell membrane uptake and internalization through permeation/diffusion, endocytosis, or receptor-mediated internalization^32^.

The facilitating effect of high glucose concentrations in uptake and apparent intracellular aggregation of αSyn in INS-1E cells sequentially treated with hIAPP and hαSyn is remarkable and suggest that cell membrane permeability and/or uptake of αSyn is facilitated at high glucose concentrations, which is intriguing given the increased risk for T2D patients in developing PD^14–17^. By provoking ER and mitochondrial stress as well as increased intracellular calcium levels, insulin resistance and ensuing hyperglycemia may enhance the probality of self- and cross-seeding of αSyn and IAPP in β-cells by increasing the levels, and thus availability, of endogenous, unfolded and misfolded IAPP and αSyn. Increased levels of αSyn has been shown to provoke both oxidative and ER stress^33,34^ and oxidative stress has also been shown to enhance cell-to-cell transfer of αSyn^34^. Insulin resistance and dysglycemia are also associated with reduced β-cell proteasome and autophagic activity^35,36^, i.e. the cellular processes that ensures clearance of misfolded proteins and aggregates, thus providing an additional mechanism by which insulin resistance and dysglycemia may lead to increased levels of misfolded and aggregating peptides.

Soluble oligomers of amyloidogenic proteins, including αSyn and IAPP, are suggested to be more toxic than the mature fibrils and to exert their toxicity in part by affecting membrane integrity^6,8,37,38^, leaving open the possibilty that also heterooligomeric aggregates may be membrane toxic. Moreover, amyloidogenic proteins have been proposed to possess the ability of a prion-like type of transmission^32^. Although the potential route(s) for a prion-like type of transmission remains largely unkown, αSyn pathology has been observed in the enteric nervous system at stages prior to any pathological changes in the brain^39^ and a gut to brain axis spread of αSyn aggregates and PD pathology via the vagal nerve has been proposed^40^. In support for a vagal nerve transmission of αSyn from the gut to the brain, vagotomy has been shown to prevent the spread of gut injected αSyn to the brain in a mouse model of Parkinson’s disease^41^ and moreover vagotomy has been found in some epidemiological studies to reduce the risk of developing PD^0^. Enteric neurons of the gut project to the pancreas and moreover the pancreas is densely innervated by the vagus nerve leaving open the possibility of αSyn transmission to the pancreas via the enteric nervous system and/or the vagal nerve. Additionally, extracellular αSyn has been reported to be present in human plasma of both control and PD patients^42^. Alike insulin, IAPP secretion is increased during insulin resistant and pre-diabetic conditions^8,43^_,_ resulting in elevated levels of circulating IAPP. Thus, the circulatory system provides an additional potential dual path of transmission for αSyn and IAPP to β-cells.

In summary, our study show that αSyn and IAPP cross-seed fibrillar growth in vitro*,* that αSyn is taken up by β-cells in an IAPP and glucose dependent manner in vitro, and that tail vein injected αSyn enhances islet amyloid formation in vivo in mice whereas IAPP amyloid formation is reduced in mice lacking endogenous αSyn. Thus, our data not only show upon a potent cross-seeding potential between αSyn and IAPP but also provide further support for the notion of a potential prion like type of transmission for αSyn. The mode(s) of a potential prion like transmission and the extent by which such a transmission may contribute to the initiation and progression of PMD such as T2D and PD will require further studies.

## Methods

### Animals

*hIAPPtg* and *Snca*^*−/−*^ mice were obtained from JAX mice (Jax #008232 and Jax #003692, respectively) and maintrained by back-cross to CBA/CaCrl (*hIAPPtg* mice) or C57BL/6J (*Snca*^*−/−*^ mice) for more than ten generations. Wild type, *Snca*^*−/−*^, *hIAPPtg* and *hIAPPtg; Snca*^*−/−*^ mice were obtained by brother sister mating in the F1 generation from *hIAPPt*g (CBA/CaCrl) and *Snca*^*−/−*^ (C57BL/6J) breeding. Animals were housed at 12:12 h light/dark cycle in a temperature/humidity controlled (22 °C/50% humidity) room and ad libitum feeding. Animal experiments were approved by the Animal Review Board at the Court of Appeal of Northern Norrland in Umeå (approval numbers A73-15 and A11-17) and conducted in accordance with Guidelines for the Care and Use of Laboratory animals.

### Mouse and human islets

Mouse islets were isolated by collagenase digestion of the pancreas^44^ and incubated overnight in RPMI 1640 medium (GIBCO #11879-0) supplemented with 11.1 mM glucose (Sigma-Aldrich #G8769), 1% fetal bovine serum (GIBCO #10500-064), 10 mM HEPES (Umeå University, Laboratory medicine), 1 mM sodium pyruvate (Gibco, #11360-039), 50 µM 2-mercaptoethanol (Gibco, #31350-010), 50 units/ml penicillin and 50 µg/ml streptomycin (Gibco, #15140-122) at 37 °C in a humidified incubator with 5% CO_2_. Human islets from donors (6 individual donors, age range 54–79 years, diabetic and undiagnosed diabetic, body mass index range 27.8–37; see Supplemental table 1) were provided through the JDRF award 31-2008-416 ECIT Islet for Basic Research program in compliance with Swedish law and the Ethical board for human research in Umeå (https://www.epn.se, Dnr: 212-97-31M). Donor data are fully anonymized and no clinical data beyond sex, age, BMI and HbA1c were available. Upon arrival, the islets were transferred to 50 ml falcon tubes and left to settle for 5 min before removal of the supernatant and addition of culture medium CMRL 1066 medium (Gibco #21530-027), 10% fetal bovine serum (Gibco #10500-064), 20 units/ml penicillin and 20 µg/ml streptomycin (Gibco, #15140-122) and 1× GlutaMAX (Gibco #35050-038). Islets were washed with culture medium 3 additional times, transferred to petri dishes and left to recover overnight in a humidified incubator at 37 °C and 5% CO_2_.

### Tail vein injections

10 weeks old *hIAPPtg* mice and wild type littermate controls were anaesthetized by intraperitoneal injection of 50 µl of Midazolam/Hypnorm (final dilution 1:4 in sterile 0.15 M NaCl, respectively). 100 µl of 7 or 70 µM hαSyn monomers, fibrils or sonicated fibrils (denoted seeds) in sterile PBS were administered by tail vein injection. Mice were surveyed during recovery and kept at normal housing conditions with free access to food and water. At 20 weeks of age, mice were sacrificed and pancreas was isolated in liquid-N_2_ and stored at − 80 °C before mounting in cryo-medium (NEG-50) on carbondioxide ice and then kept at − 80 °C until amyloid analyses.

### Amyloid analyses

Amyloid content quantified as total Thioflavin-S^+^ amyloid area over total islet area by staining isolated pancreases with Thioflavin-S as previously described^45^. Fluorescent images were acquired with a Nikon Eclipse E800 fluorescence microscope, using a 20 × Plan Fluor DIC M ∞/0.17 WD objective. Islets amyloid was quantified in pancreases isolated from 20 weeks old *hIAPPtg* and wild type littemates that were tail-vein injected at 10 weeks of age with PBS (n = 161 islets/15 wt mice and n = 159 islets/17 *hIAPPtg* mice) serving as controls for mice injected with either 7 µM hαSyn monomers (59 islets/6 wt mice and 154 islets/16 *hIAPPtg* mice), 7 µM hαSyn fibrils (151 islets/15 wt mice and 230 islets/24 *hIAPPtg* mice), 7 hαSyn µM seeds (120 islets/12 wt mice and 216 islets/22 *hIAPPtg* mice),respectively, or for mice injected with 70 µM hαSyn monomers (60 islets/6 wt mice and 42 islets/4 *hIAPPtg* mice), 70 µM hαSyn seeds (70 islets/7 wt mice and 62 islets/6 *hIAPPtg* mice), and 70 µM hαSyn fibrils (71 islets/7 wt-mice and 61 islets/6 *hIAPPtg* mice), respectively, was. Similarly, islets amyloid quantification was done on pancreatic tissues isolated from 10 and 20 weeks old wild type (57 islets/5 mice; 10 weeks old), *Snca*^−/−^ ( 58 islets/6 mice; 10 weeks old), *hIAPPtg* (100 islets/12 mice; 10 weks old and 144 islets/13 mice; 20 weeks old)*,* and *hIAPPtg; Snca*^*−/−*^*,* mice (71 islets/9 mice; 10 weeks old and 125 islets/12 mice; 20 weeks old).

### Amyloid/fibril isolation

Mouse pancreases were perfused using collagenase type XI (Sigma-Aldrich, C7657) and incubated for 15 min at 37 °C to degrade collagen fibrils, homogenized for 15 s at 25,000 rpm (Polytron PT1300D, Kinematica AG, Swizerland) in buffer A (10 mM TRIS–HCL pH 7.4, 0.25 M sucrose, 3 mM EDTA, one Roche Complete Protease Inhibitor tablet per 50 ml, 0.1% NaN_3_), and rotated overnight at 4 °C. Solid sucrose was added to a final concentration of 1.2 M and the homogenate was centrifugated at 170,000 × *g*, 4 °C for 30 min. The top layer, cellular debris, and intermediate supernatant were discarded. The remaining sample was resuspended in buffer A with 1.9 M sucrose, mixed thoroughly and centrifugated at 125,000 × *g*, for 30 min, 4 °C. The solid top layer was collected, transferred to a new tube, resuspended in 20 ml 50 mM TRIS buffer pH 8.0 and centrifugated at 8000 × *g*, 15 min, 4 °C. The supernatant was removed and the pellet resuspended in 50 mM TRIS pH 8.0, 2 mM CaCl_2_, 0.01 mg/ml DNAse1, incubated for 3 h at room temperature, and centrifugated at 8000 × *g*, 15 min, 4 °C. The supernatant was removed and the pellet was resuspended in 50 mM TRIS pH 8.0 and centrifugated at 8000 × *g*, 15 min, 4 °C. The supernatant was removed and the resulting pellet was resuspended in 50 mM TRIS pH 8.0, 1.3 M sucrose, 1% SDS and immediately centrifugated at 170,000 × *g*, 4 °C for 45 min. The pellet was kept and 50 mM TRIS pH 8.0 added to the supernatant to reduce the sucrose concentration followed by centrifugation at 170,000 × *g*, 4 °C for 45 min. Both pellets were combined and washed two times in milliQ (to remove traces of sucrose) and centrifugated twice at 170,000 × *g*, 4 °C for 45 min. The pellet was resuspended in 100 μl PBS with 0.1% NaN_3_ and stored at − 80 °C. The above described procedure, omitting the collagenase perfusion step, was used also for amyloid/fibril isolation from human islets.

### Tokuyashu technique for immunolabeling

Isolated islets were fixed in 2% paraformaldehyde (PFA) (EM grade; Fisher Scientific, PA0095, batch 76336), 0.2% glutaraldehyde (GA) (25% stock, EM grade; Taab Laboratories Equipment Ltd, G011/2, batch no 24680) in 0.1 M phosphate buffer (PB) pH 7.4, 4 °C overnight. The solution was replaced with freshly prepared 1% PFA in 0.1 M PB pH 7.4 and stored at + 4 °C. The fixated islets were washed at least three times in 0.1% glycine (Merck, 56-40-6) in PBS followed by incubation for 10 min at room temperature in 0.1% glycine in PBS and the solution was replaced with 12% gelatine (Dr. Oetker) in 0.1 M PB, pH 7.4, and incubated at 37 °C for 30 min. The islets were subsequently transferred to a fresh 12% gelatine solution and incubated for additional 10 min at 37 °C, briefly centrifugated, and the tube with pelleted islets were placed on ice. The part of the sample containing islets was cut out, and transferred to 2.3 M sucrose overnight at 4 °C. Next day the islets were mounted to metal pins facing the surface with an extra drop of 2.3 M sucrose, to prevent ice crystal formation upon freezing, and instantly frozen in liquid nitrogen. To prepare cryosections the sample was trimmed and the presence of the islets was confirmed under a light microcospe. Ultra thin sectioning of approximately 70 nm thickness was done using an Ultra microtome (Leica EM UC7) and the sections were placed on copper grids 200 mesh, with a formvar film and the carbon layer of approximately 3 nm.

For immunogold labelling, the grids with tissue sections were placed facing a 2% gelatin (Dr Oetker) in 0.1 M PBS, pH 7.4 for 5 min at room temperature and then for 20 min at 37 °C. The grids were washed 3 × 2 min in 0.1% glycine in 0.1 M PBS, pH 7.4, and blocked for 5 min in 1% gelatine blocking buffer (1% gelatine from cold water fish [Sigma-Aldrich, G7041, lot # SLBN4365V] in 0.1 M PBS, pH 7.4). anti-αSyn antibodies (pan-αsyn: sc-7011-R, Santa Cruz; human specific αsyn: sc-12767, Santa Cruz) or anti-IAPP antibodies (NBP-1-06579, Novus Biologicals, USA) diluted 1:50, respectively, in 1% gelatine blocking buffer were added to the grids and incubated for 45 min. The grids were washed five times in 1% gelatine blocking buffer for 2 min and incubated with Protein-A-Gold (Cell Microscopy Core, Univeristy Medical Center Utrecht, The Netherlands) diluted 1:25–50 (batch depended) in 1% gelatine blocking buffer, for 20 min. The grids were washed in 0.1 M PBS, pH 7.4 five times for 2 min. Fixation was performed with 1% glutaraldehyde in 0.1 M PBS, pH 7.4, for 5 min and followed by six consecutive washes in milliQ for 1 min. Contrasting was done by incubating the grids in 2% uranyloxaalacetate, pH 7. The grids were briefly washed in methyl-cellulose/uranyl acetate, pH 4, on ice and incubated for 5 min to avoid drying artefacts. The grids were looped out using handmade Remanium wire loops and imaged on the transmission electron microscope (JEOL JEM-1230, Japan).

For double immune gold labelling, grids were sequentially stained with two different primary antibodies where the first primary antibody was detected by 10 nm Protein-A-Gold following the protocol described above all the way down to fixation with 1% glutaraldehyde. After glutaraldehyde fixation step, grids were washed five times in 1 × PBS and then blocked a second time for 5 min in 1% cold water fish gelatine blocking buffer before continuing to stain the grids with the second primary antibody detected with 15 nm Protein-A-Gold particle following the protocol above. After glutaraldehyde fixation and the following washing step grids were contrasted, dried and looped out as described above before analyses in the transmission electron microscope.

### Antibodies

See supplemental table 2.

### Peptides

hαSyn was purchased from rPeptide (catalog #S-1001-2), dissolved in phosphate saline (PBS), pH 7.4 (Gibco, # 10010-023) and filtered using a 0.2 μm filter (Millex-GV, #SLGV013SL). hIAPP was obtained from Calbiochem (catalog #05-23-2540) and dissolved in hexafluoroisopropanol (Sigma-Aldrich, # 105228) to completely dissolve hIAPP. Before cell culture experiments (see below) hexafluoroisopropanol, which is toxic to cells was evaporated under nitrogen gas to remove the solvent and hIAPP was then reconstituted in culture medium to the desired concentration. rIAPP was purchased from AnaSpec Inc. (catalog # AS-60253-1), dissolved in phosphate saline (PBS), pH 7.4 (Gibco, # 10010-023) and filtered using a 0.2 μm filter (Millex-GV, # SLGV013SL).

### Fibril formation assay

PBS buffer pH 7.4, 9 mM NaN_3_, was used for the aggregation experiments. hαSyn seeds were prepared by sonicating aggregated hαSyn (i.e. the end product formed in fibril formation reaction described below) with a 10% amplitude and a pulse of 0.5 s followed by 0.5 s break (Branson Digital Sonifier 450; Branson Ultrasonics Tapered 1/8" Microtip probe ). All reactions were performed in a total volume of 100 μl in 96 well plates (Corning, ref. 3650) with a 3-mm glass beads and incubated at 37 °C and orbital shaking (300 rpm) in a ClarioStar (Eppendorf) in presence of 20 μM ThT (Sigma, T3516-5G, lot# MKBZ3617V). ThT was excited at 418 nm and the emission spectra was recorded from 477 to 497 nm until ThT fluorescent the signal plateaud, which was used as an indicator of the protein aggregation state. Fluorescence spectra of 20 μM ThT in solution were subtracted from each sample measurement. ThT fluorescence detected aggregation curves were made by plotting the emission signals on y-axis vs the aggregation time on x-axis. All ThT curves are averages of at least six replicates and all experiments were performed at three independent times, apart from the cross-seeding with lower 0.2 μM αSyn species (Supplementary Fig. 8) that were averages of five replicates performed at four independent times.

### Cell culture and treatment

Rat insulinoma INS-1E cells were purchased from AddexBio Technologies. Cells were cultured in RPMI 1640 (Gibco, #21875-034) supplemented with 10% fetal bovine serum (Gibco, #10500-064), 10 mM HEPES (Umeå University, Laboratory medicine), 1 mM sodium pyruvate (Gibco, #11360-039), 50 µM 2-mercaptoethanol (Gibco, #31350-010), 50 units/ml penicillin and 50 µg/ml streptomycin (Gibco, #15140-122) at 37 °C in a humidified incubator with 5% CO_2_. Passage 40–55 were used for all experiments. The cells were seeded (100,000 cells/well) on Poly-l-lysine precoated coverslips (Sigma-Aldrich, P4832) in 24-well plates (Corning). 48 h after plating, the cells were washed once with PBS, and then fresh medium supplemented with 11 or 22 mM glucose containing 5 µM monomeric hIAPP was added to the cells. After 4 h of incubation, the cells were washed twice with PBS to remove non-bound monomers. Each well were replaced with 400 µl of fresh RPMI 1640 medium containing 1 µM monomeric hαsyn and incubated for an additional period of 20 h. Alternatively, INS-1E cells were incubated for 24 h with RPMI 1640 (11 or 22 mM glucose) containing 1 µM monomeric hαsyn.

### Immunofluorescence

Following treatment with hIAPP and hαsyn, INS-1E cells were washed once with PBS and fixed with 4% formaldehyde for 15 min at room temperature (RT). The cells were washed three times with PBS and then permebilized with 0.15% Triton X-100 in PBS for 15 min at RT. Cells were blocked for 60 min with 3% BSA in PBS and incubated with primary antibodies in 3% BSA/PBS overnight at 4 °C. The cells were washed three times with PBS and subsequently incubated with Alexa-conjugated secondary antibodies for 1 h at RT. Finally, the cells were rinsed three times in PBS and coverslips were mounted with ProLong Diamond Antifade reagent containing DAPI (Invitrogen, P36966). For information on antibodies used, see Supplemental Table 2.

### Confocal microscopy

Fluorescence images were acquired using a Nikon A1 confocal laser microscope (Nikon), using a 60 × PlanApo/1.4-NA oil-immersion objective. The pinhole was adjusted to keep the same size of z-optical sections for all channels. Images were acquired using the NIS-Elements acqusition software (Nikon). Images were assembled using Adobe Photoshop software (Adobe Systems Co.). To quantify hαsyn^+^ cytoplasmic inclusions in the INS-1E cells, five to eight randomly selected fields from each experiment were imaged. Serial *z*-stacks were acquired in 1-µm increments and the total number of cells containing inclusions from the middle two images from each z-stack was counted. From this, the percentages of cells with positive inclusions were calculated. Pooled data from three independent experiments were statistically analyzed using the unpaired Students *t*-test.

### Quantification and statistical analyses

Quantification of amyloid content and αsyn^+^ insclusions was performed using Image-J software (version1.49 m). All the statistical analyses of in vitro and mouse in vivo data were performed by two-tailed Students t-tests. We considered a value of p < 0.05 to be statistically significant.

## Supplementary information

Supplementary Information.

## Data Availability

All data generated and/or analysed during this study are either included in this article (and its Supplementary information) or are available from the corresponding author on reasonable request.
