# Supplementary material for: α-Synuclein promotes IAPP fibril formation in vitro and β-cell amyloid formation in vivo in mice
Source: Sci Rep. 2020 Nov 24;10:20438. doi: 10.1038/s41598-020-77409-z (PMC7686322; doi:10.1038/s41598-020-77409-z)
Supplement: Supplementary file 1 — Supplementary Information. [file 41598_2020_77409_MOESM1_ESM.docx]

Supplemental information

α-Synuclein promotes IAPP fibril formation in vitro and β-cell amyloid formation in vivo in mice

Authors: Marija Mucibabic, Pär Steneberg, Emmelie Lidh, Jurate Straseviciene, Agnieszka Ziolkowska, Ulf Dahl, Emma Lindahl, and Helena Edlund*

*Corresponding author. E-mail: helena.edlund@umu.se

1. Supplemental figures

2. Supplemental tables

3. Supplemental methods


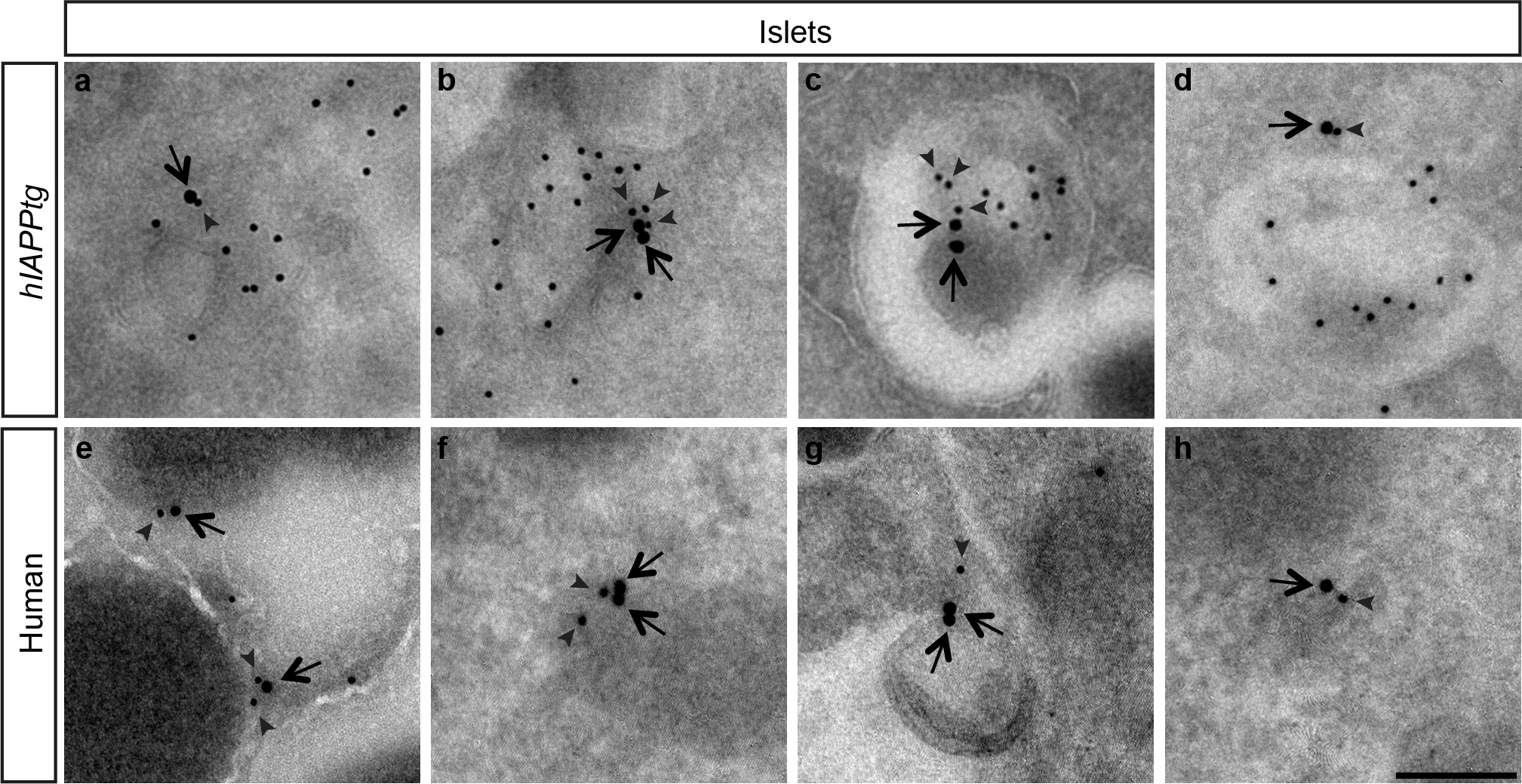


**Figure S1. αSyn and IAPP co-localize in mouse and human β-cells.** (**a-h**) TEM images of *hIAPPtg* mouse islets isolated from 4 independent mice (**a-d**) and human islets isolated from separate donors (**e-h**) (donor #4, 1, 2, and 3, from left to right) showing β-cells immuno-gold labelled for αSyn (sc-7011R, 15 nm gold particles) and IAPP (NBP1-06579, 10 nm gold particles). Scale bar is 200 nm in **a-h.**

**
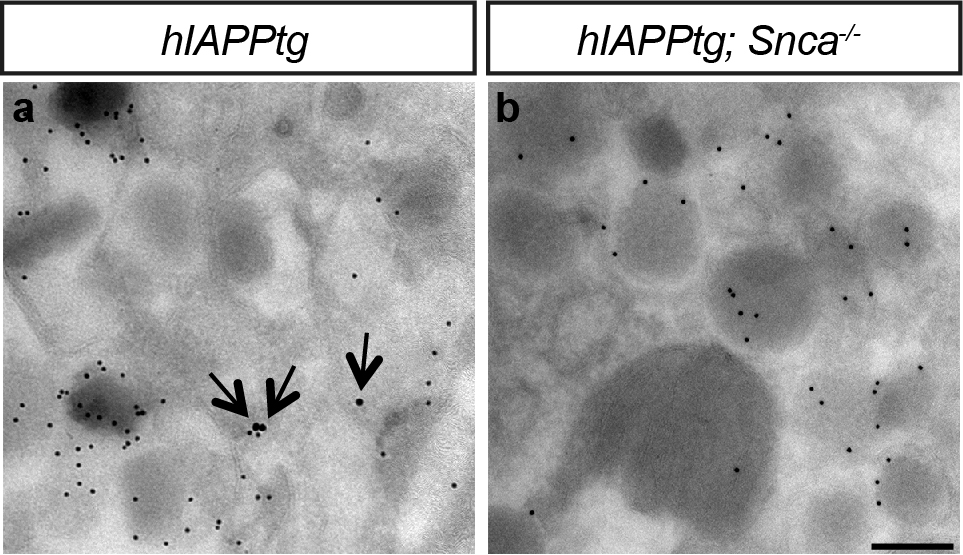
**

**Figure S2. αSyn antibody SC-7011R specificity in mouse β-cells.** (**a-b**) TEM images of *hIAPPtg* and *hIAPPtg; Snca^-/-^* mouse islets β-cells immuno-gold labelled for αSyn (sc-7011R, 15 nm gold particles) and IAPP (NBP1-06579, gold 10 nm gold particles), demonstrating lack of αSyn immunoreactivity in β-cells of *hIAPPtg* mice deficient for αSyn, i.e. *hIAPPtg; Snca^-/-^* mice. Black arrows indicate αSyn gold particles. Scale bar is 200 nm in **a, b**.

**
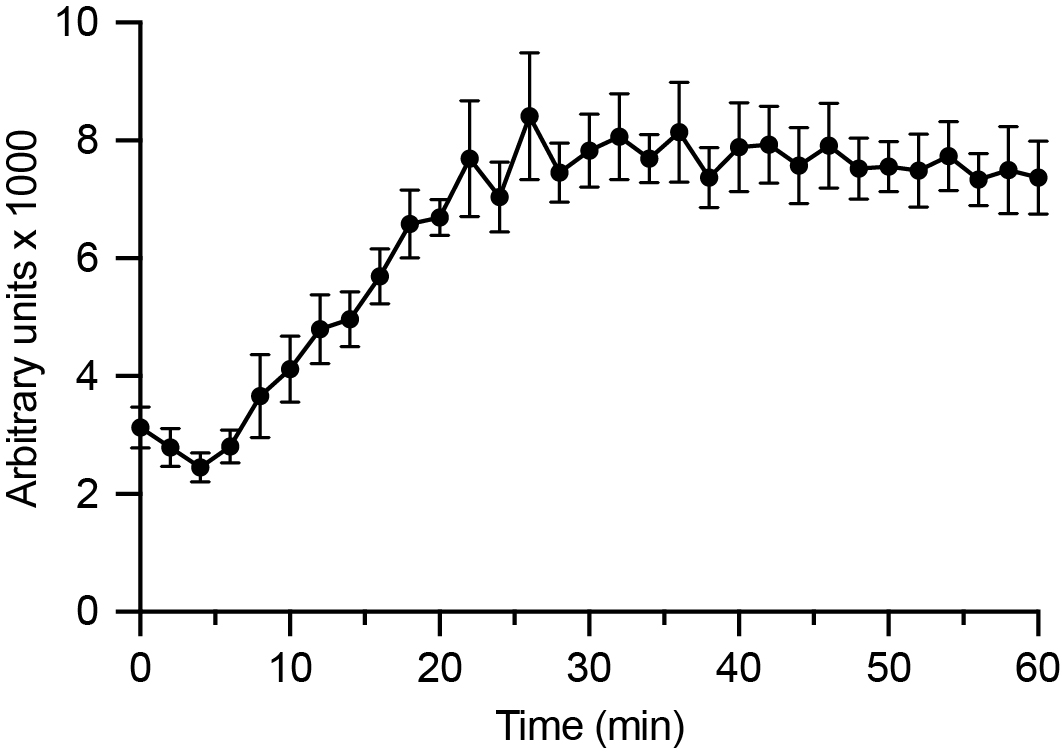
**

**Figure S3. ThT curve for hIAPP monomer homo-seeding.** Close up 2 µM hIAPP monomer fibril formation from Fig. 2a showing the lag phase. Data are presented as mean value +/- SEM.


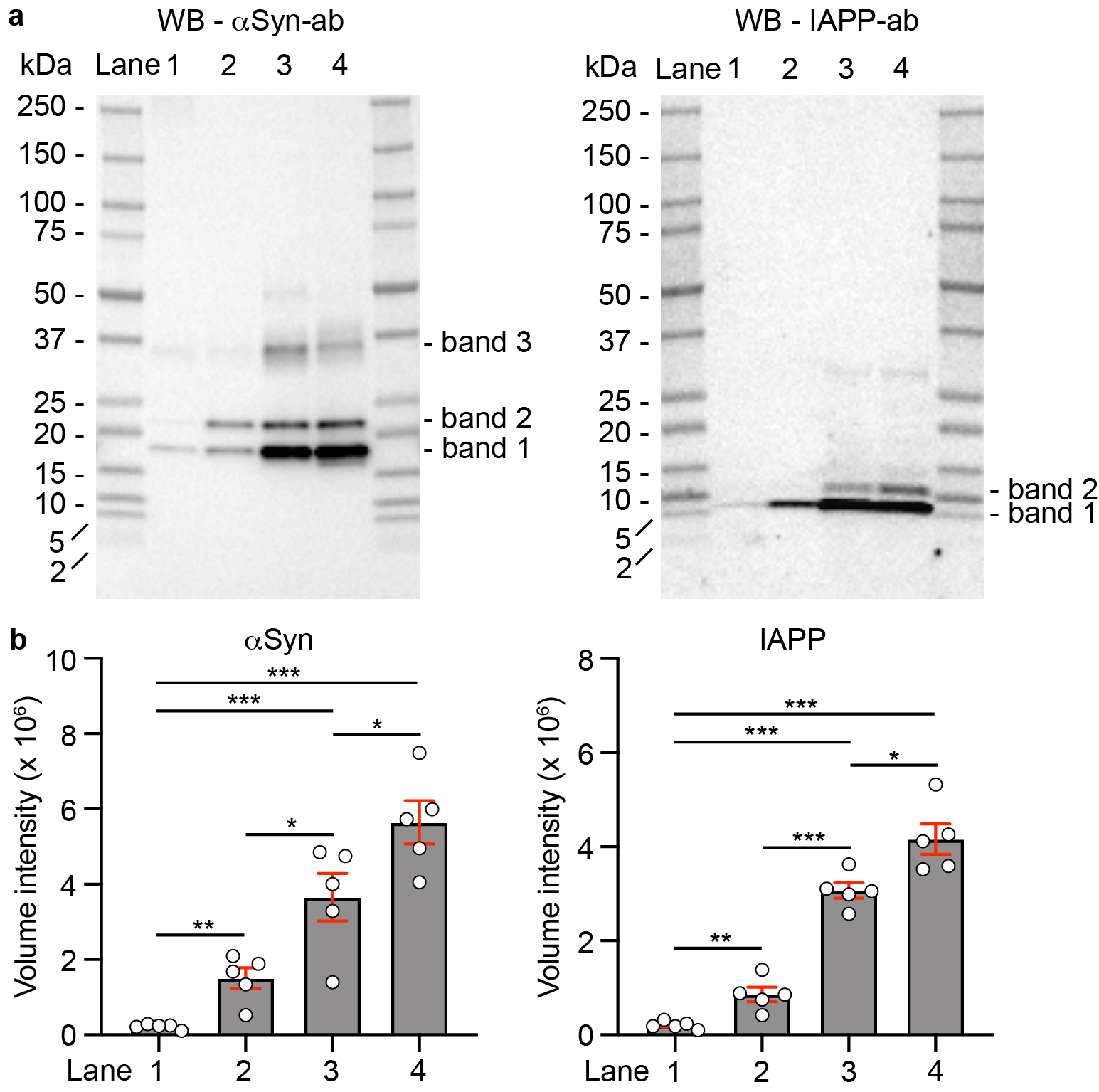


**Figure S4. Western blot analyses of fibrillar growth. (a-b)** Representative immunoblots **(a)** and quantification **(b)** of the amount of αSyn (bands 1-3 pooled) and IAPP (bands 1 and 2 pooled) in the pelleted fibrils formed at the first lag phase (lane 1), first growth phase (lane 2), second lag phase (lane 3), and end stage plateau (lane 4), n=5 for all timepoints, when cross-seeding 2 µM hIAPP monomers with 7 µM hαSyn monomers. Data are presented as mean +/- SEM, **P* < 0.05, ***P* < 0.01, ****P* < 0.001 (Student’s t-test).

**
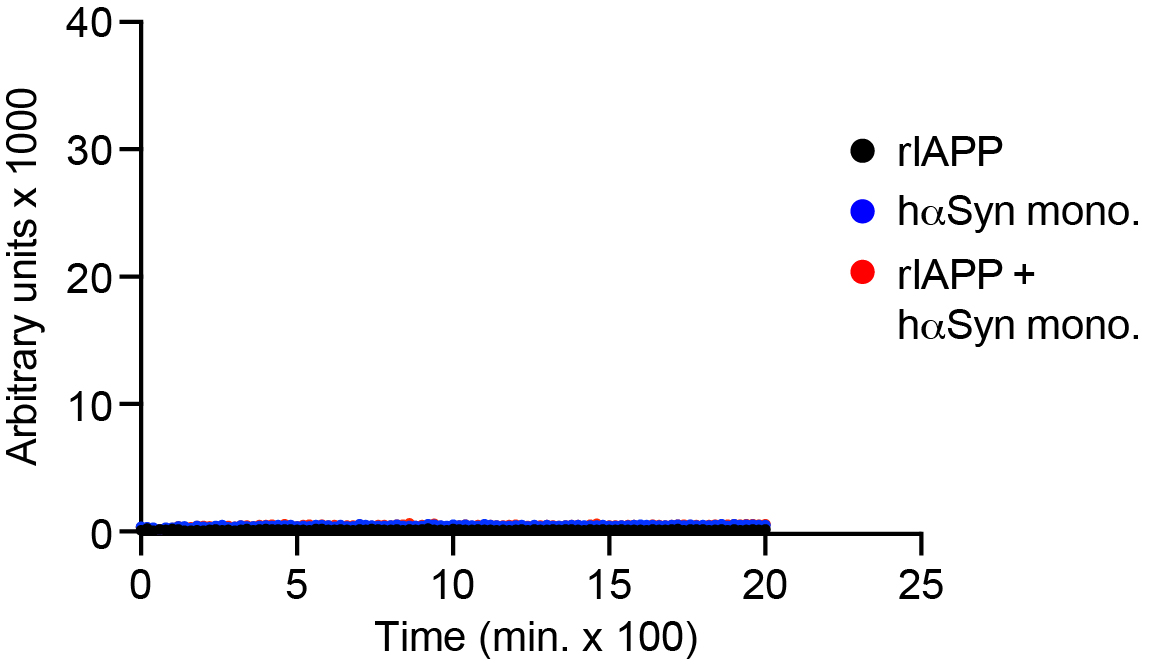
**

**Figure S5**. **rIAPP and aSyn monomer co-incubation.** Fibril formation ThT curves for 2 µM rat IAPP (rIAPP) monomers alone (black), 7 µM hαSyn monomers alone (blue), and 2 µM rIAPP monomers with 7 µM αSyn monomers (red). Data are presented as mean value +/- SEM.

**
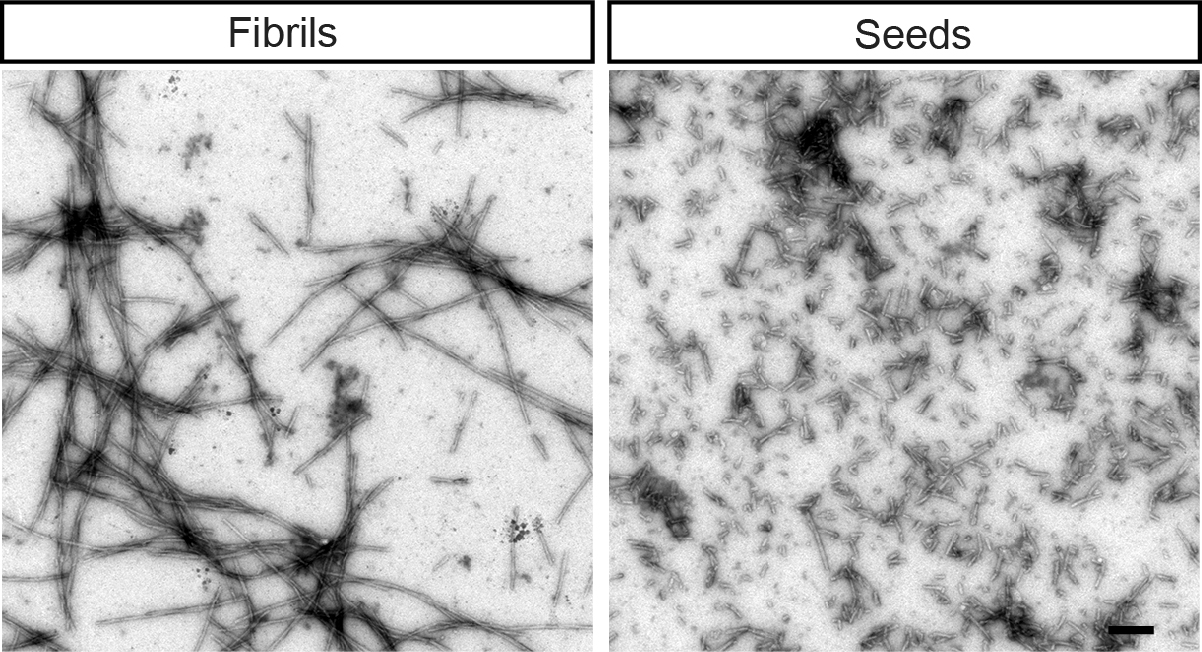
**


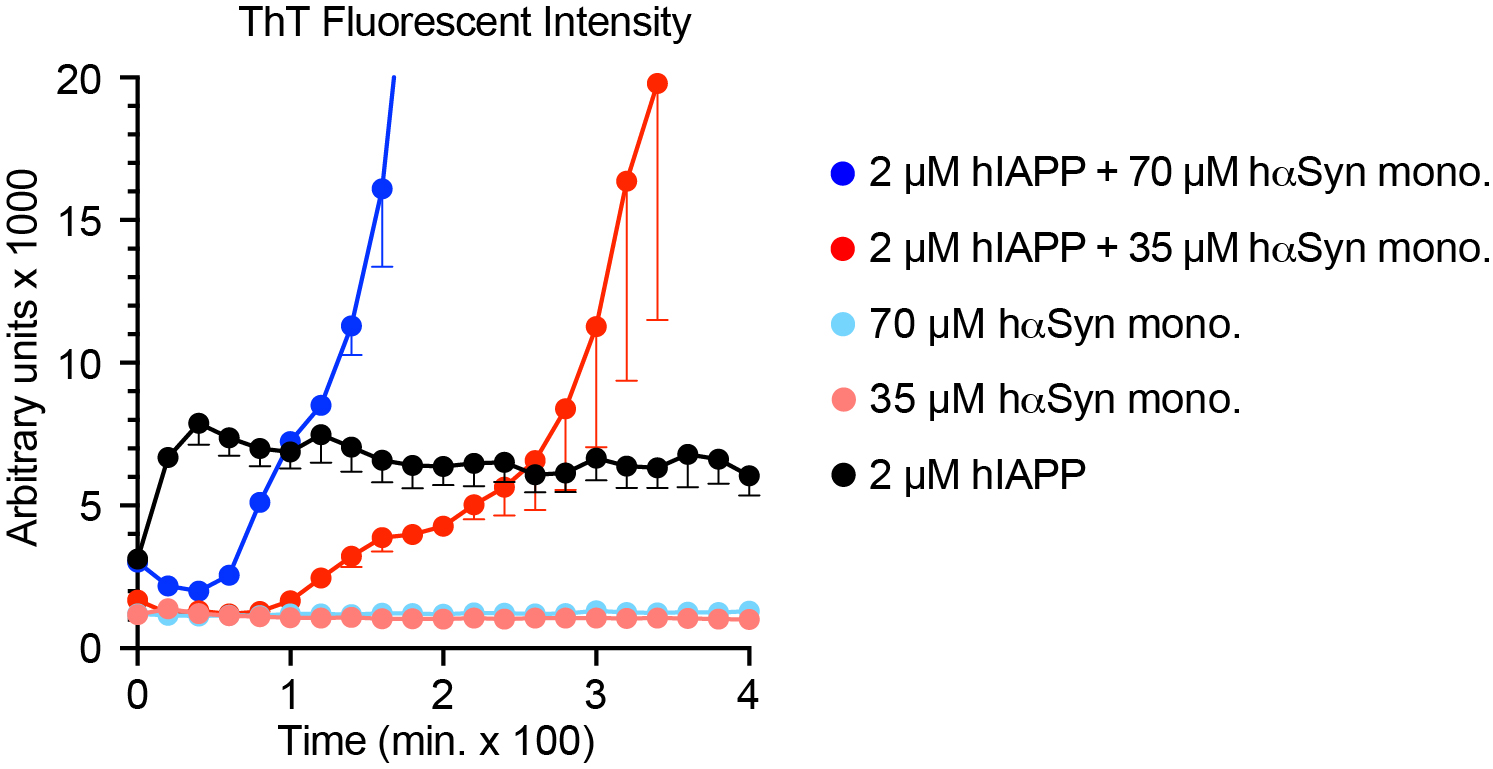


**Figure S6. αSyn fibrils and seeds.** TEM negative stain images of fibrils (left) formed *in vitro* from hαSyn monomers and hαSyn seeds (right) obtained by sonication of *in vitro* formed hαSyn fibrils. Scale bar is 200 nm.

**
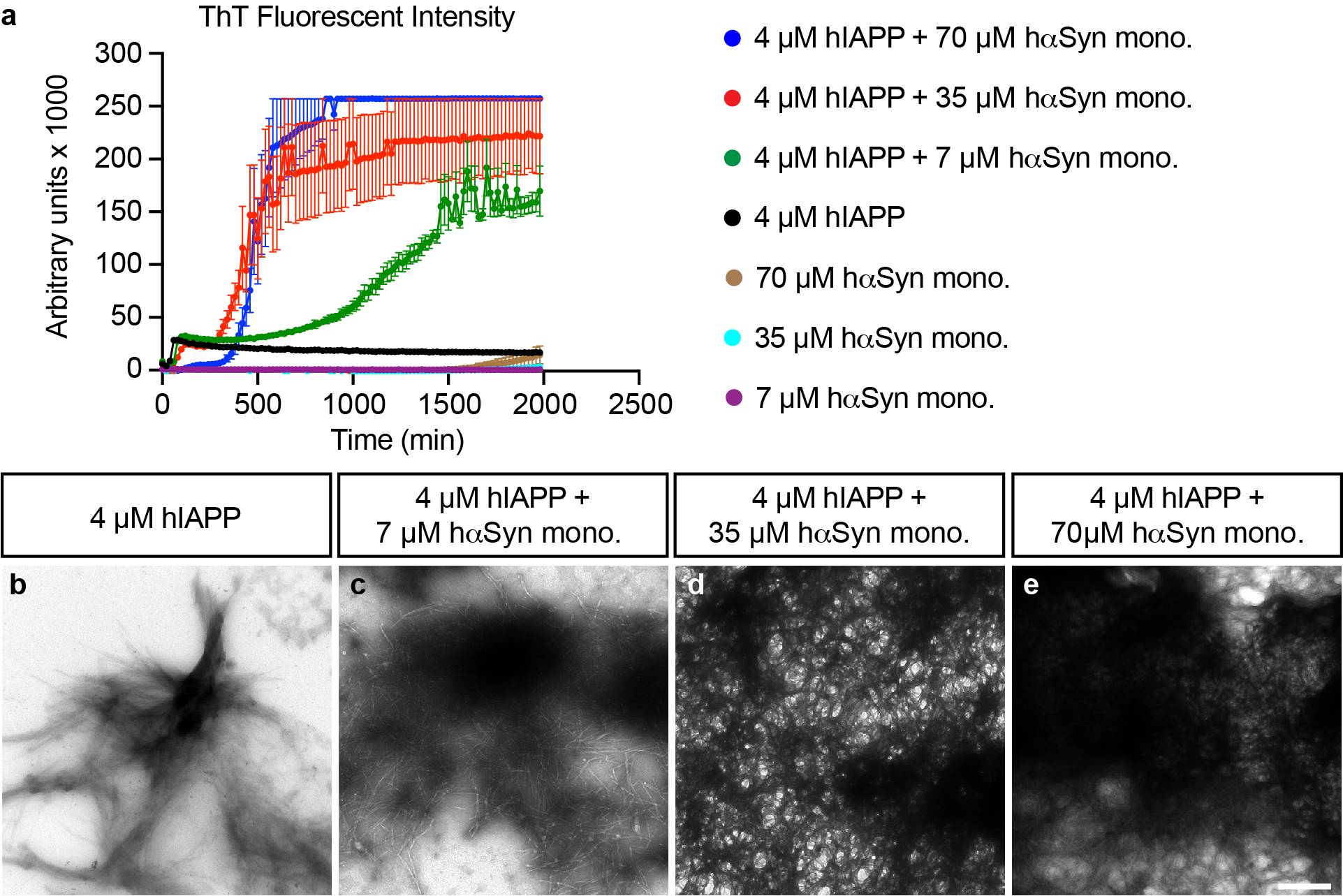
**

**Figure S7. Close up of ThT curves for 35 and 70 µM αSyn monomer cross-seeding of IAPP monomers.** Fibril formation ThT curves for the first 400 minutes co-incubation of 2 µM hIAPP monomers with 35 µM (red) and 70 µM hαSyn monomers (blue), respectively. Shown are also curves for 2 µM hIAPP monomers alone (black) and 35 µM (pink) and 70 µM (light blue) hαSyn monomers alone. Data are presented as mean value +/- SEM.

**Figure S8. αSyn monomers dose-dependently cross-seed 4 µM IAPP monomers.** (**a**) Fibril formation ThT curves for 4 µM hIAPP monomers alone (black), hαSyn monomers alone in concentrations of 7 µM (purple), 35 µM (light blue), and 70 µM (brown), respectively, and combinations of 4 µM hIAPP monomers with 7 µM (green), 35 µM (red), and 70 µM (blue) hαSyn monomers, respectively. Please note that the ThT emission exceeds the setting of the plate reader when co-incubating 4 µM hIAPP monomers with 70 µM hαSyn monomers (blue curves in **a**), hence the maximal ThT emissions for these reactions are likely larger than that displayed in **a.** Data in a are presented as mean value +/- SEM. (**b-e**) TEM negative stain images of the resulting fibrils formed in (**a**); 4 µM hIAPP alone (**b**), 4 µM hIAPP with 7 µM (**c**), 35 µM (**d**), and 70 µM (**e**) hαSyn, respectively. Scale bar is 400 nm in **b-e**.


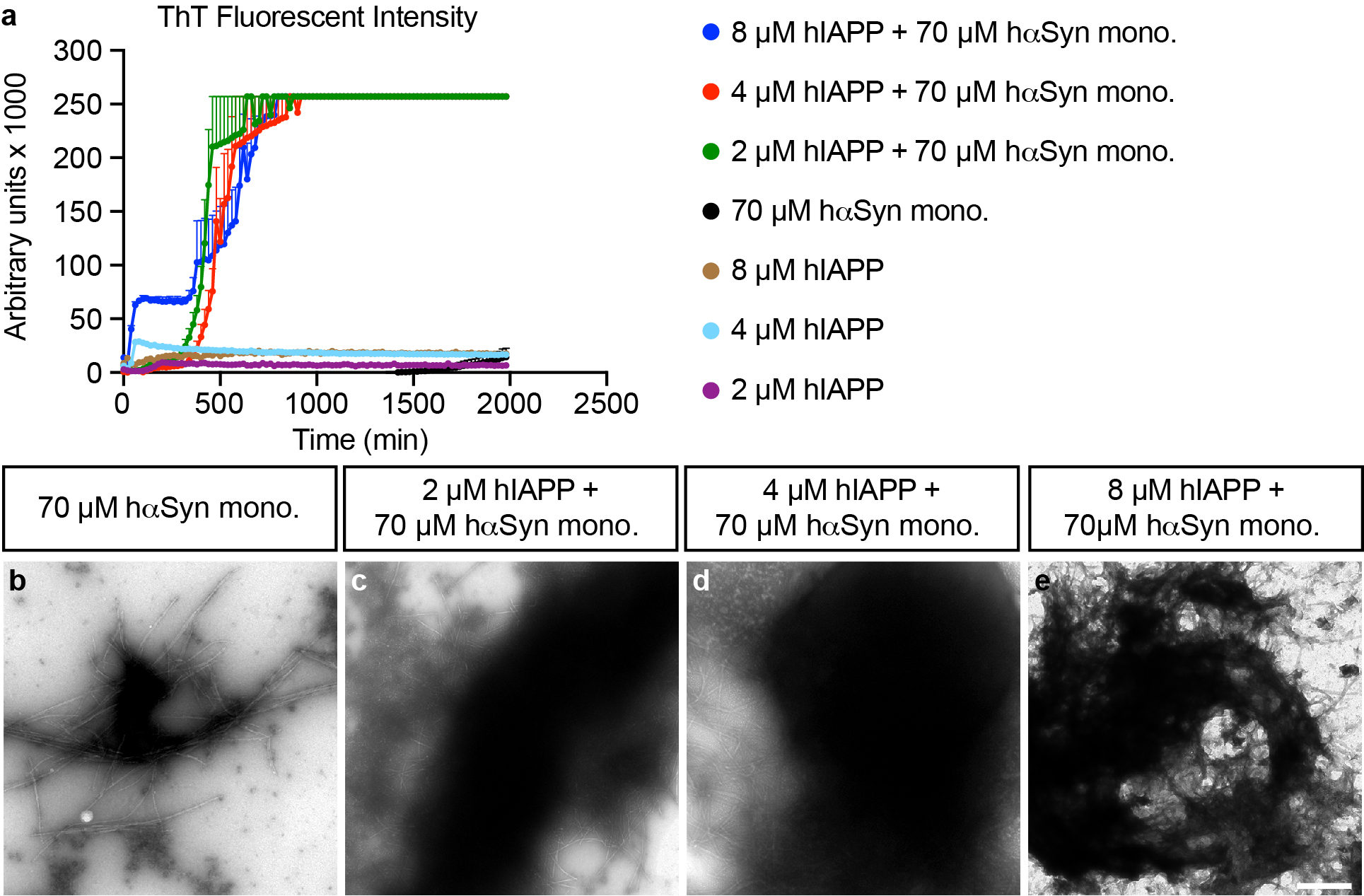


**Figure S9. IAPP monomers cross-seed αSyn monomers.** (**a**) Fibril formation ThT curves for 70 µM hαSyn monomers alone (black), hIAPP monomers alone in concentrations of 2 µM (purple), 4 µM (light blue), and 8 µM (brown), respectively, and combinations of 70 µM hαSyn monomers with 2 µM (green), 4 µM (red), and 8 µM (blue) hIAPP monomers, respectively. Please note that the ThT emission exceeds the setting of the plate reader when co-incubating 70 µM hαSyn monomers with 2, 4, and 8 µM hIAPP monomers (green, red, and blue curves in **a**), hence the maximal ThT emissions for these reactions are likely larger than that displayed in **a.** Data in a are presented as mean value +/- SEM. (**b-e**) TEM negative stain images of the resulting fibrils formed in (**a)**; 70 µM αSyn alone (**b**), 70 µM hαSyn with 2 µM (**c**), 4 µM (**d**), and 8 µM (**e**) hIAPP, respectively. Scale bar is 400 nm in **b-e**.

**
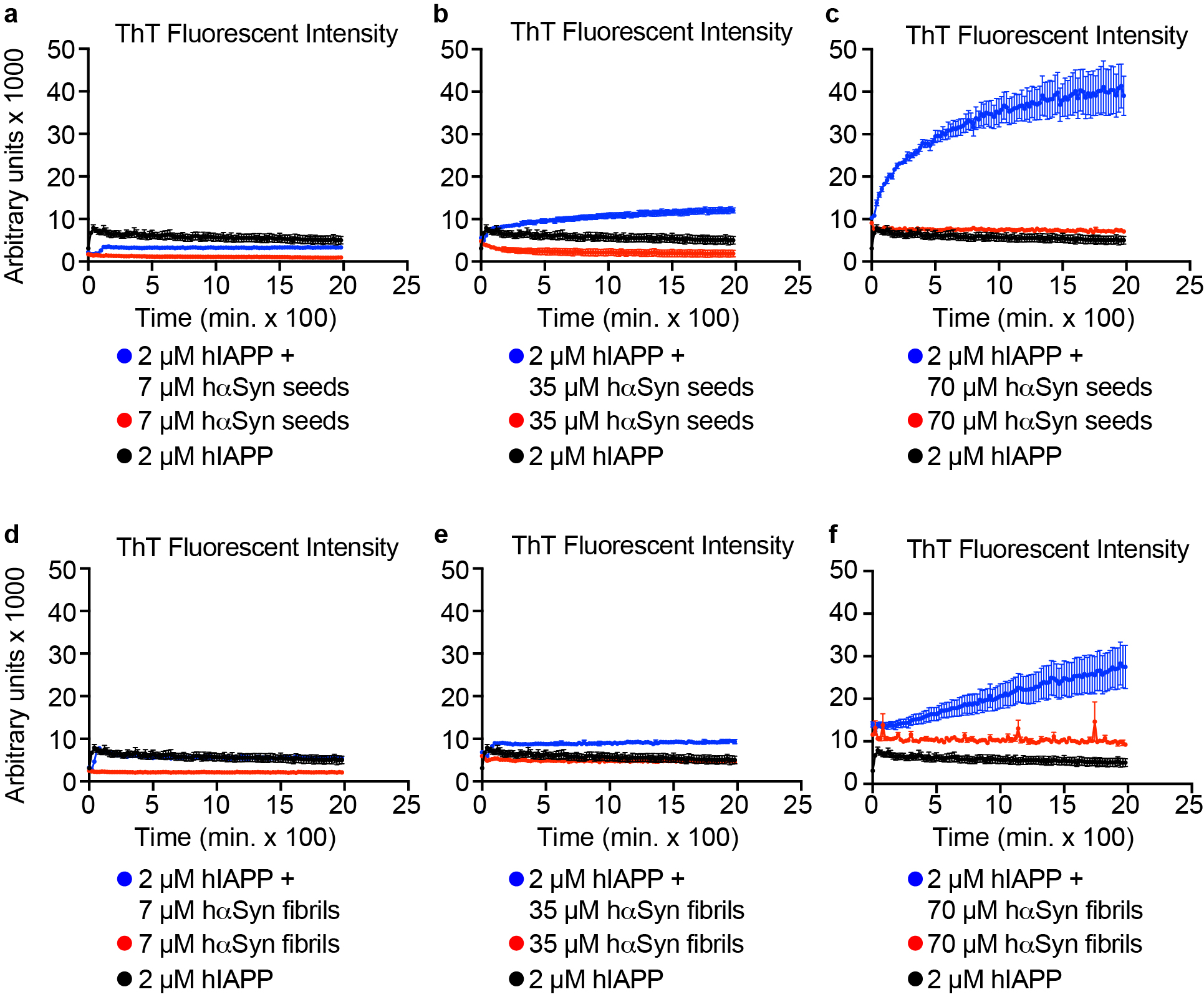
**

**Figure S10. Cross-seeding of IAPP monomers with increasing concentrations of αSyn seeds and fibrils.** (**a-c**) Fibril formation ThT curves for 2 µM hIAPP monomers alone (black in **a-c**), 7, 35, and 70 µM hαSyn seeds alone (red in **a-c**), and 2 µM hIAPP monomers with 7, 35, and 70 µM hαSyn seeds (blue in **a-c**). (**d-f**) Fibril formation ThT curves for 2 µM hIAPP monomers alone (black in **d-f**), 7, 35, and 70 µM µM hαSyn fibrils alone (red in **d-f**), and 2 µM hIAPP monomers with 7, 35, and 70 µM hαSyn fibrils (blue in **d-f**). Data are presented as mean value +/- SEM.

**
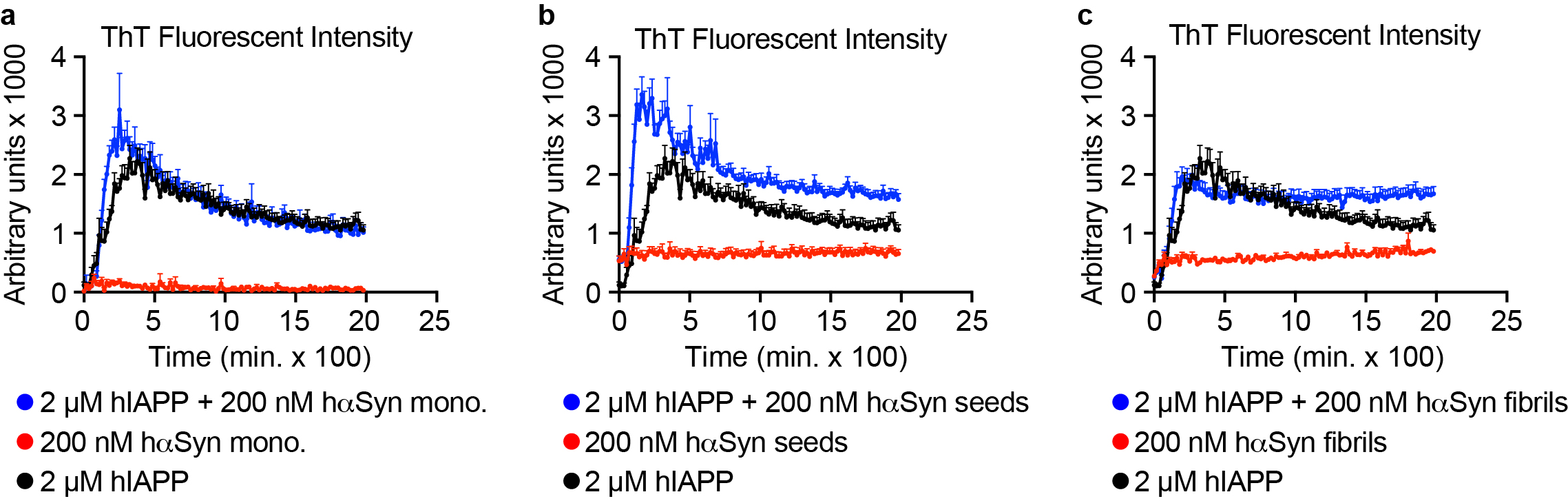
**

**Figure S11. Cross-seeding of 2** **µM IAPP with 0.2 µM αSyn monomers, seeds, and fibrils.** (**a-c**) Fibril formation ThT curves for 2 µM hIAPP monomers alone (black in **a-c**), 0.2 µM hαSyn monomers (**a**), seeds (**b**), and fibrils (**c**) alone (red in **a-c**) and 2 µM hIAPP monomers with 0.2 µM hαSyn monomers (**a**), seeds (**b**), and fibrils (**c**) (blue in **a-c**). Data are presented as mean value +/- SEM.

**Supplemental table 1. Human islets donors**

|  | Age (years) | Gender | BMI (kg/m2) | HbA1c | SI |
| --- | --- | --- | --- | --- | --- |
| Donor #1 | 61 | Male | 37 | 53 mmol/mol | 2.8 |
| Donor #2 | 79 | Female | 27.8 | 54 mmol/mol | 4.8 |
| Donor #3 | 54 | Male | 31.1 | 44 mmol/mol | 1.3 |
| Donor #4 | 62 | Male | 30.9 | 47 mmol/mol | 9.9 |
| Donor #5 | 63 | Male | 27.8 | 38 mmol/mol | 7.0 |
| Donor #6 | 55 | Male | 30.5 | 46 mmol/mol | 6.4 |

**Supplemental table 2. Antibodies**

|  | Antigen | Species | Supplier |
| --- | --- | --- | --- |
| Primary antibodies | pan-αsyn | Rabbit | Santa Cruz (cat. nr. 7011R) |
|  | αsyn (human specific) | Mouse | Santa Cruz (cat. nr. 12767) |
|  | IAPP | Rabbit | Novus Biologicals  (cat. nr. 06579) |
|  | Syntaxin | Rabbit | Santa Cruz (cat nr. 13994) |
|  | Amylin/IAPP | Rabbit | Peninsula (cat. nr. T4157) |
|  | αsyn | Rabbit | Abcam (cat nr. ab138501) |
| secondary antibodies | Alexa Fluor 594 Plus conjugated anti-Mouse IgG | Goat | Invitrogen (cat.nr. A-32742) |
|  | Alexa Fluor 488 conjugated anti-Rabbit IgG | Donkey | Invitrogen (cat.nr. A-21206) |
|  | peroxidase-conjugated Anti-Rabbit IgG (H+L) | Goat | Jackson Laboratories (cat.nr. 111-035-003) |

**Supplemental methods**

**Western blot analyses of fibrillar aggregates**

Aggregating fibrils (100 μL) were retrieved from 96 well plates at different timepoints of incubation in the ClarioStar (Eppendorf). The aggregates were centrifuged at 10,000 g for 30 minutes at 4°C. The supernatant was transferred to a clean microcentrifuge tube and 100 μL of PBS was added to the remaining pellet and vortexed to resuspend the pellet. 33 μL of 4x sample buffer was added to each tube of resuspended pellet and supernatant. The samples were vortexed and then incubated at 100 °C for 10 min and allowed to cool for 5 min on ice before separation on 4-15% Tris-glycine SDS-PAGE gels. The gel was then blotted onto a nitrocellulose filter that was then boiled in PBS for 5 minutes. The filter was blocked in TBST with 5% BSA for 1hr and then incubated with α-syn (Abcam; ab138501) and IAPP (Peninsula; T4157) overnight at 4°C. Reactivity was visualized with peroxidase conjugated secondary antibodies and chemiluminescence detection. Using global analyses in the Image Lab software from BioRad, total amounts of pelleted fibrils were quantified by densitometric analyses of bands identified with anti-α-syn and anti-IAPP antibodies, respectively.
